# HLA-A, -B, -C, -DRB1, -DQA1, and -DQB1 allele and haplotype frequencies defined by next generation sequencing in a population of East Croatia blood donors

**DOI:** 10.1038/s41598-020-62175-9

**Published:** 2020-03-26

**Authors:** Stana Tokić, Veronika Žižkova, Mario Štefanić, Ljubica Glavaš-Obrovac, Saška Marczi, Marina Samardžija, Katerina Sikorova, Martin Petrek

**Affiliations:** 1 Department of Medical Chemistry, Biochemistry and Clinical Chemistry, Faculty of Medicine, University of Osijek, J. Huttlera 4, HR-31000 Osijek, Croatia; 2 Department of Nuclear Medicine and Oncology, Faculty of Medicine, University of Osijek, J. Huttlera 4, HR-31000 Osijek, Croatia; 3 Department of Laboratory Diagnostics and Clinical Transfusion Medicine, Clinical Institute of Transfusion Medicine, Osijek University Hospital, J. Huttlera 4, HR-31000 Osijek, Croatia; 4 Department of Pathological Physiology, Faculty of Medicine and Dentistry, Palacký University, Hnevotinska 3, 775 15 Olomouc, Czech Republic

**Keywords:** Immunogenetics, Genetics research

## Abstract

Next-generation sequencing (NGS) is increasingly used in transplantation settings, but also as a method of choice for in-depth analysis of population-specific HLA genetic architecture and its linkage to various diseases. With respect to complex ethnic admixture characteristic for East Croatian population, we aimed to investigate class-I (HLA-A, -B, -C) and class-II (HLA-DRB1, -DQA1, -DQB1) HLA diversity at the highest, 4-field resolution level in 120 healthy, unrelated, blood donor volunteers. Genomic DNA was extracted and HLA genotypes of class I and DQA1 genes were defined in full-length, -DQB1 from intron 1 to 3′ UTR, and -DRB1 from intron 1 to intron 4 (Illumina MiSeq platform, Omixon Twin algorithms, IMGT/HLA release 3.30.0_5). Linkage disequilibrium statistics, Hardy-Weinberg departures, and haplotype frequencies were inferred by exact tests and iterative Expectation-Maximization algorithm using PyPop 0.7.0 and Arlequin v3.5.2.2 software. Our data provide first description of 4-field allele and haplotype frequencies in Croatian population, revealing 192 class-I and class-II alleles and extended haplotypic combinations not apparent from the existing 2-field HLA reports from Croatia. This established reference database complements current knowledge of HLA diversity and should prove useful in future population studies, transplantation settings, and disease-associated HLA screening.

## Introduction

**Figure 1:**
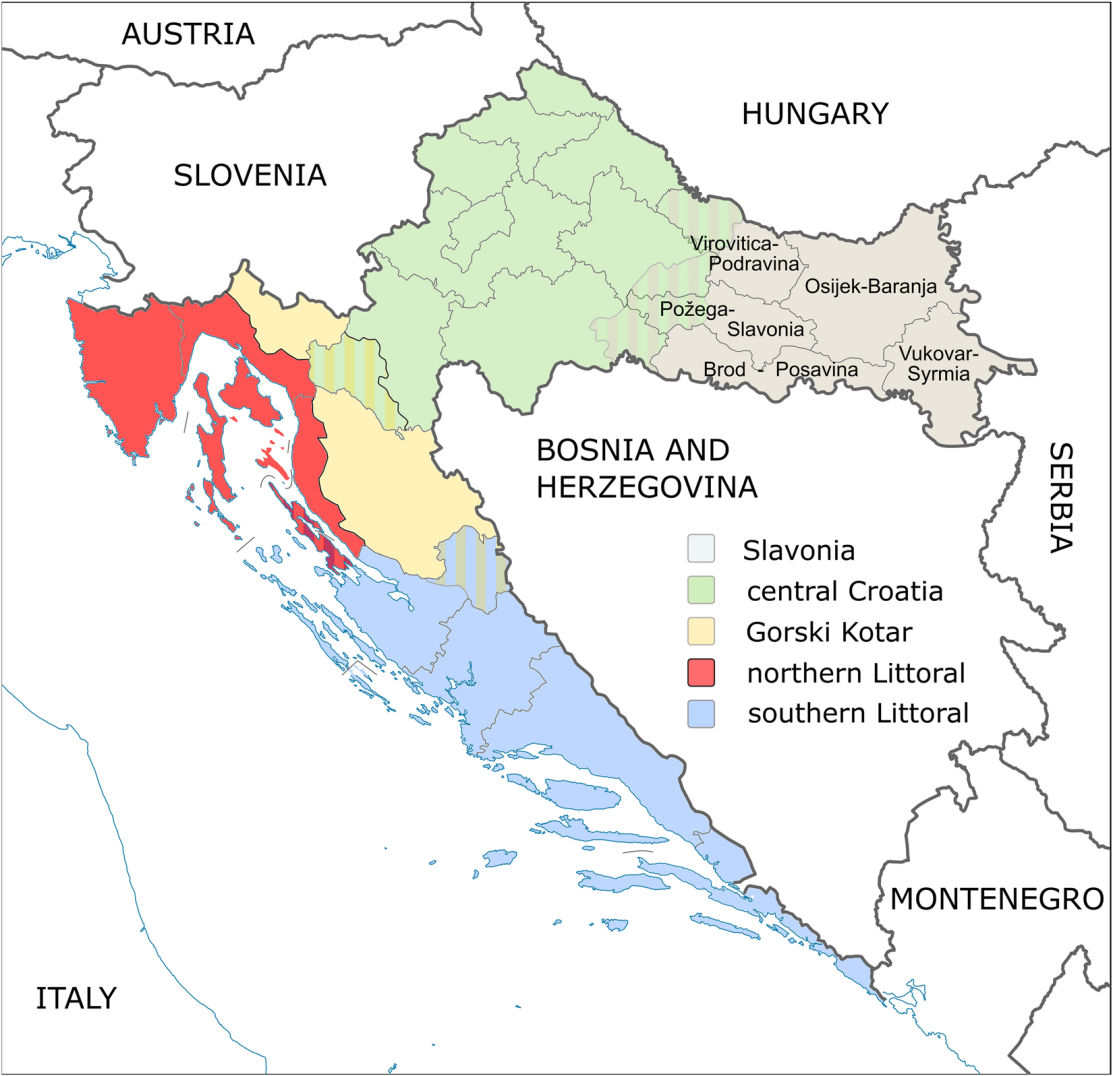
Map of geographical location of Croatia with representation of Dalmatia, Istria, Gorski Kotar, central and eastern Croatian regions, five of which (colored in light grey) participated in sample collection. In detail: Osijek-Baranja county (n = 80), Vukovar-Syrmia county (n = 22), Brod-Posavina county (n = 9), Požega-Slavonia county (n = 4) and Virovitica-Podravina county (n = 5)^56^.

Croatia is a Mediterranean, crescent-shaped south European country bordering Slovenia in the northwest, Hungary in the northeast, Serbia in the east, Bosnia and Herzegovina and Montenegro in the southeast, and Italy along the maritime border. Croatia consists of three major geomorphologic areas, which can be further broken down into five traditional districts based on history, topography, and economy; Istria and Dalmatia in the northern and southern Croatian littoral, Gorski Kotar in country’s mountainous area, central continental Croatia, and Slavonia in the Pannonian basin in the east (Fig. 1). Slavonia territory was originally populated by the southern branch of the Indo-European Slavic populations in the 7^th^ century^1^, and has been a witness of significant population admixture ever since, including the Hungarian migration to Slavonia in 10^th^ century, and the influx of Islamic and Orthodox Balkan and Asian populations during the Ottoman conquest in 16^th^ century, causing at the same time, the continuous shift of Catholics from Bosnia to Slavonia during several centuries^2^. Under the auspices of Habsburg monarchy, the settlement of Germans and Austrians in Slavonian urban areas peaks between 18^th^ and 19^th^ century, while Orthodox Vlachs from Bosnia, immigrating Czechs, Slovaks, Ukrainians, Italians, and Croatians from Gorski Kotar populate rural settlements^3^. These historic and more recent 20^th^ century migration events, encompassing emigration of Germans and Austrians from Slavonia, and the settlement of Balkan War veterans from Serbia, Croatian immigrants from Dalmatia, Herzegovina, and most recently also from north Bosnia, have shaped the genetic diversity of East Croatian population.

Population migration and admixture have an important role in the evolution and diversifying selection of hypervariable human leukocyte antigen (HLA) molecules involved in innate and adaptive immune responses^4^. Two types of HLA molecules, class I and class II, are codominantly expressed on the surface of nucleated and antigen presenting cells, and facilitate peptide antigen presentation, self-tolerance and immune surveillance through activation of CD8 and CD4 T lymphocytes, respectively. The biological function of HLA molecules is correlated with high variability of human major histocompatibility (MHC) proteins, which are encoded by the highly polymorphic gene complex located in the short arm of chromosome 6^5^. Up to date, 18.691 class I, 7.065 class II and 202 non-HLA allelic variants (IPD-IMGT/HLA database, release 3.38., https://www.ebi.ac.uk/ipd/imgt/hla/stats.html)^6,7^ have been identified in various human populations and the highest degree of variability was noticed within exon regions encoding phenotype and peptide-binding preferences of class I (HLA-A, -B, -C) and class II (HLA-DR, -DQ) HLA molecular isotypes.

Considering important role of HLA molecules in immune response, the HLA gene complex has been extensively studied in the context of allogeneic transplantation, inflammatory, infectious and autoimmune disease associations^8^. The worldwide application of molecular, PCR-based techniques in the clinical settings (sequence-specific primer (SSP), sequence-specific oligonucleotide (SSO), Sanger sequencing-based typing (SBT) and most recently, next-generation sequencing (NGS)), also enabled development of the population specific HLA typing data repositories (The Allele Frequency Net Database)^9^, and enhanced assessments of populations migration, diversity and regional HLA specificity. Significant differences in frequency of common and well documented alleles recently described within European sub-regions, support the role of geographical dispersion in development of region specific HLA heritage^10^. The inter- and intra-population HLA comparisons made today are however, mostly constrained to a partial, exon description of HLA genetic variability (1^st^ and 2^nd^ fields of HLA nomenclature), whereas synonymous (3^rd^ field) and non-coding (4^th^ field) nucleotide variations remain largely unexplored. In such settings, it remains challenging to accurately build contiguous long haplotypes of uniformly high resolution even for the largest sample cohorts. The implementation of NGS technologies in HLA research and routine clinical work, enables however, elucidation of full-length HLA gene sequences, permitting an in-depth characterisation of population HLA diversity. In this context NGS can overcome limitations of traditional typing techniques, thereby sustaining optimal HLA matching of donor-recipient pairs for organ and particularly, haematopoietic stem cell transplantation (HSCT)^11^, improved estimates of population structure and HLA associated disease risk^12–14^, and better understanding of demographic history and geographic origin of a given population^15^.

Up to date, HLA allelic and haplotype diversity of a general Croatian population, irrespective of specific geographical preferences, was estimated in several large cohorts originating from Croatia^16–19^ and emigrant population in Germany^20^, as well as few, more isolated populations from particular geographical locations such as island Krk^21^, island Hvar^22^, Istrian city of Rijeka^23^ and Gorski Kotar^24^. Moreover, several non-frequent, rare and very rare HLA-A, -B and -DRB1 alleles and haplotypes have been characterized among the unrelated volunteer donors from the Croatian Bone Marrow Donor Registry (CBMDR)^25^. In addition, DPB1 allelic diversity was recently evaluated in 82 Croatian patients who underwent HSCT^26^. These previous studies were however, based on lower resolution (1^st^ and 2^nd^ field) HLA typing of selected HLA loci in individuals originating from various Croatian regions, providing partial insight into the HLA diversity of a general, but not East Croatia population.

The aim of the present study was thus to investigate and describe extended allelic and haplotype diversity of HLA-A, -B, -C, -DQA1, -DQB1 and DRB1 loci at high-resolution, 4^th^ field level, using high-throughput NGS technique for HLA typing of 120 healthy, unrelated blood donor volunteers from east Croatia.

## Results

### NGS sequencing results

**Figure 2:**
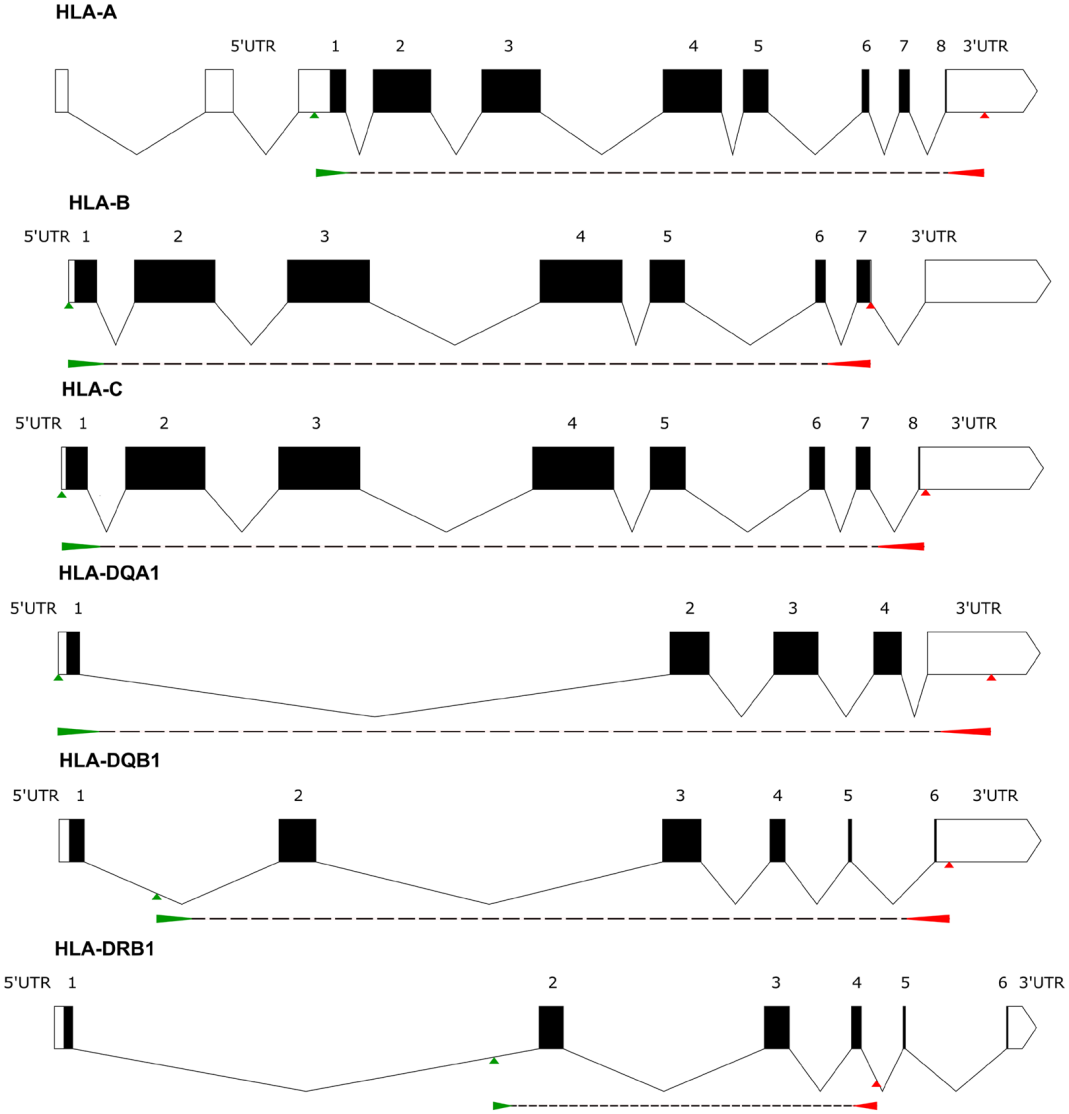
Shematic representation of HLA-A, -B, -C, -DQA1, -DQB1 and -DRB1 gene regions covered by Omixon Holotype PCR primers are depicted using Exon-Intron Graphic Maker v.4. (http://wormweb.org/exonintron)^57^. Start positions of foreward and reverse primers are marked by green and red arrows, respectively.

We evaluated 120 donors (120 donors × 6 loci × 2 alleles = 1440 alleles), 9 of whom were excluded from further analysis due to low-performing samples on quality control check [low read count (≤2500 bp for class I and ≤5000 bp for class II genes) and/or low key exon coverage depth (≤30)]. The coverage of each locus in the remaining samples (111 donors, 1332 alleles) was calculated by Twin as the percentage of gene regions covered by reads compared to the whole allele sequence (coverage %). The Omixon Holotype primer positioning allowed only partial amplification of 3′UTRs and 5′UTRs, and covered exons 2–6 of DQB1 and exons 2–4 of DRB1 loci (Fig. 2).

Ambiguous allele calls, which remained unresolvable due to inherent product limitations of the Omixon Holotype Kit (missing data on SNPs or INDEL variations within the unsequenced 3′ UTR, 5′ UTR and intron 1/exon 1 regions), were reported as ambiguous (i.e. DQB1*06:01:01/15), or up to the third field level only (i.e. DQB1*05:03:01), and are enlisted in Supplementary Table 1. Nevertheless, all amplified exon/intron regions of each allele in the remaining samples were fully covered (detection %), with an average coverage depth of >140 reads per nucleotide position (Supplementary Table 2). On average, 54359 (Supplementary Table 3) high-quality reads were produced per sample, of which 91% were subsequently used for final consensus generation after removing noise reads and PCR crossover artefacts. The average fragment size was 259 bp, and the average read length 208 bp.

### Linkage disequilibrium and HWE estimates

**Figure 3:**
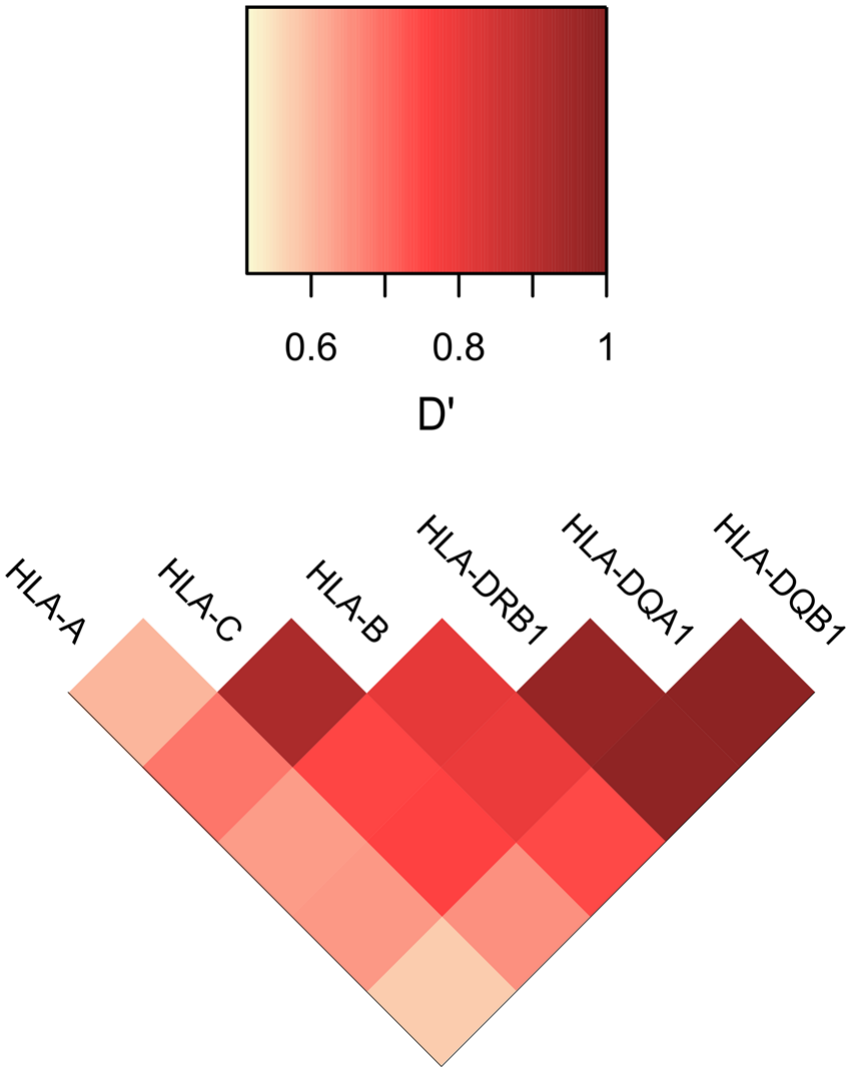
Heat map of HLA-A~B~C~DRB1~DQA1~DQB1 linkage disequilibrium (LD) expressed in terms of the D’ measure. The upper colour-key represents the range of D’. Bright yellow colours represent lower D’ values, while dark red colours demonstrate high haplotype LD. R (v3.6.0), packages: gplots, RColorBrewer (http://www.R-project.org)^58^.

Genotype frequencies of HLA-A, -B, -C, -DRB1, -DQA1 and DQB1 loci did not deviate from Hardy-Weinberg expectations (Supplementary Table 4). Strong linkage was confirmed between class I, HLA-A, -B, and -C, as well as class II, HLA-DQA1, -DQB1 and DRB1 loci (Supplementary Table 5 and Fig. 3).

### Class I allelic frequencies

The observed frequencies of class I HLA alleles in eastern Croatian population are reported in Table 1. HLA-A genotyping analysis uncovered 25 different alleles in our sample population, five of which with frequency ≥10%. The most commonly observed HLA-A*02:01:01 group alleles (32.43%) were followed by HLA-A*01:01:01:01 (12.16%), HLA-A*03:01:01:01 (11.71%), HLA-A*24:02:01:01 (11.71%) and HLA-A*11:01:01:01 (9.91%). Together, these allelic variants comprised 77.47% of HLA-A allelic diversity in our sample population. Nearly half of the tested individuals (n = 59) were positive for HLA-A*02:01:01 allelic variants, 13 of which were homozygous carriers.

**Table 1:** HLA-A, HLA-B and HLA-C allele (AF) and cumulative frequencies (cF) in the sample of 111 unrelated blood donor volunteers from East Croatia.

	Allele HLA-A	n	AF	cF	Allele HLA-B	n	AF	cF	Allele HLA-C	n	AF	cF
1	A*02:01:01	72	32.43%	32.43%	B*51:01:01	19	8.56%	8.56%	C*04:01:01:01	31	13.96%	13.96%
2	A*01:01:01:01	27	12.16%	44.59%	B*08:01:01	18	8.11%	16.67%	C*07:01:01	25	11.26%	25.23%
3	A*03:01:01:01	26	11.71%	56.31%	B*35:01:01	18	8.11%	24.77%	C*02:02:02	21	9.46%	34.68%
4	A*24:02:01:01	26	11.71%	68.02%	B*07:02:01	15	6.76%	31.53%	C*07:02:01	18	8.11%	42.79%
5	A*11:01:01:01	22	9.91%	77.93%	B*35:03:01	15	6.76%	38.29%	C*12:03:01	16	7.21%	50.00%
6	A*32:01:01:01	8	3.60%	81.53%	B*18:01:01	12	5.41%	43.69%	C*06:02:01:01	15	6.76%	56.76%
7	A*23:01:01:01	5	2.25%	83.78%	B*15:01:01:01	9	4.05%	47.75%	C*03:03:01	12	5.41%	62.16%
8	A*25:01:01:01	5	2.25%	86.04%	B*27:05:02	9	4.05%	51.80%	C*01:02:01	11	4.96%	67.12%
9	A*31:01:02:01	5	2.25%	88.29%	B*44:02:01:01	9	4.05%	55.86%	C*05:01:01	11	4.96%	72.07%
10	A*26:01:01:01	4	1.80%	90.09%	B*27:02:01:01	8	3.60%	59.46%	C*07:04:01	11	4.96%	77.03%
11	A*30:01:01	3	1.35%	91.44%	B*52:01:01	8	3.60%	63.06%	C*04:01:01:06	9	4.05%	81.08%
12	A*68:01:02:02	3	1.35%	92.79%	B*55:01:01	8	3.60%	66.67%	C*12:02:02	8	3.60%	84.69%
13	A*24:02:01:05	2	0.90%	93.69%	B*57:01:01:01	7	3.15%	69.82%	C*15:02:01:01	7	3.15%	87.84%
14	A*29:02:01:01	2	0.90%	94.59%	B*13:02:01:01	6	2.70%	72.52%	C*03:04:01	5	2.25%	90.09%
15	A*66:01:01:01	2	0.90%	95.50%	B*44:27:01	6	2.70%	75.23%	C*07:01:02	4	1.80%	91.89%
16	A*03:01:01:03	1	0.45%	95.95%	B*44:03:01	5	2.25%	77.48%	C*08:02:01:01	3	1.35%	93.24%
17	A*03:01:01:05	1	0.45%	96.40%	B*15:17:01:01	4	1.80%	79.28%	C*14:02:01	3	1.35%	94.60%
18	A*26:01:01:06	1	0.45%	96.85%	B*35:02:01	4	1.80%	81.08%	C*07:18	2	0.90%	95.50%
19	A*26:08	1	0.45%	97.30%	B*38:01:01	4	1.80%	82.88%	C*15:05:02	2	0.90%	96.40%
20	A*29:01:01:01	1	0.45%	97.75%	B*39:01:01	4	1.80%	84.69%	C*17:03:01	2	0.90%	97.30%
21	A*31:01:02:04	1	0.45%	98.20%	B*14:02:01:01	3	1.35%	86.04%	C*03:02:02:01	1	0.45%	97.75%
22	A*33:01:01:01	1	0.45%	98.65%	B*40:01:02	3	1.35%	87.39%	C*03:04:02	1	0.45%	98.20%
23	A*33:03:01:02	1	0.45%	99.10%	B*40:02:01	3	1.35%	88.74%	C*06:02:01:02	1	0.45%	98.65%
24	A*68:01:01:02	1	0.45%	99.55%	B*41:02:01	3	1.35%	90.09%	C*07:01:09	1	0.45%	99.10%
25	A*68:02:01:01	1	0.45%	100.00%	B*58:01:01	3	1.35%	91.44%	C*16:01:01	1	0.45%	99.55%
26					B*18:03	2	0.90%	92.34%	C*16:02:01	1	0.45%	100.00%
27					B*35:08:01:01	2	0.90%	93.24%				
28					B*37:01:01:01	2	0.90%	94.14%				
29					B*49:01:01	2	0.90%	95.05%				
30					B*56:01:01	2	0.90%	95.95%				
31					B*07:05:01	1	0.45%	96.40%				
32					B*07:06:01	1	0.45%	96.85%				
33					B*15:01:06	1	0.45%	97.30%				
34					B*15:10:01	1	0.45%	97.75%				
35					B*27:02:01:04	1	0.45%	98.20%				
36					B*44:02:01:03	1	0.45%	98.65%				
37					B*44:05:01	1	0.45%	99.10%				
38					B*50:01:01:01	1	0.45%	99.55%				
39					B*54:01:01	1	0.45%	100.00%				

As expected, the highest polymorphism was observed within the HLA-B region, encompassing 39 different allelic specificities. The HLA-B*51:01:01 (8.56%), HLA-B*35:01:01 (8.11%) and HLA-B*08:01:01 (8.11%) were the most frequent alleles, and together with the HLA-B*07:02:01 (6.76%), HLA-B*35:03:01 (6.76%) and HLA-B*18:01:01 (5.41%) accounted for majority (43.7%) of HLA-B allelic diversity in our population. Notably, HLA-B*15 (gene frequency, 6.75%), HLA-B*35 (17.57%) and HLA-B*44 (9.91%) allelic families exhibited the highest variability, each encompassing at least 4 allelic members with different peptide binding motifs. Of interest, two rare alleles (http://www.allelefrequencies.net, Rare allele detector, score<4) of undefined C/WD status were also uncovered, namely HLA-B*15:01:06 (0.45%) and HLA-B*44:02:01:03 (0.45%).

The HLA-C genotyping uncovered 26 different allelic specificities within 13 allelic families. The most polymorphic was HLA-C*07 group with 6 alleles, covering 27.48% of HLA-C allelic variability. Moreover, 50 subjects, were positive for either HLA-C*07:01:01 G (14.41%) or HLA-C*07:02:01 G (8.11%) allelic variants^6,7^. Nonetheless, the most common individual HLA-C representative was the HLA-C*04:01:01:01 (13.96%) allele, followed by HLA-C*02:02:02 (9.46%), HLA-C*12:03:01 (7.21%) and HLA-C*03:03:01 (5.41%). Rare, but well-documented HLA-C*07:01:09 allele of undefined C/WD status was also found.

### Class II allelic frequencies

Detailed analysis of class II HLA alleles is presented in Table 2. Among class II genes, DRB1 exhibited greatest allelic diversity through 40 alleles, of which HLA-DRB1*16:01:01 (13.51%) and HLA-DRB1*01:01:01 (10.81%) were most commonly present (>10%). Of interest, HLA-DRB1*16:01:01 allele was twice as frequent as the HLA-DRB1*15:01:01 (6.76%), or HLA-DRB1*03:01:01:01 (5.41%). Together with HLA-DRB1*07:01:01 (9.91%) and HLA-DRB1*11:01:01:01 (4.5%), these 6 DRB1 alleles were responsible for 50.9% of DRB1 allelic variability. Notably, HLA-DRB1*16 (13.96%) and HLA-DRB1*11 (13.5%) were the most common allelic families in our donor population. One of the DRB1*11 family members was a rare DRB1*11:01:08 allele, spotted only once. The highest individual variability, however, was detected within the DRB1*04 allelic group, which was comprised out of 9 different allelic members, together covering 8.11% of DRB1 allelic variability in our population.

**Table 2:** HLA-DRB1, HLA-DQA1 and HLA-DQB1 allele (AF) and cumulative frequencies (cF) in the sample of 111 unrelated blood donor volunteers from East Croatia.

	Alelle HLA-DRB1	n	AF	cF	Allele HLA-DQA1	n	AF	cF	Allele HLA-DQB1	n	AF	cF
1	DRB1*16:01:01	30	13.51%	13.51%	DQA1*01:02:02	35	15.77%	15.77%	DQB1*05:02:01:01	35	15.77%	15.77%
2	DRB1*01:01:01	24	10.81%	24.32%	DQA1*03:01:01	16	7.21%	22.97%	DQB1*03:01:01:03	33	14.86%	30.63%
3	DRB1*07:01:01	22	9.91%	34.23%	DQA1*02:01:01:01	15	6.76%	29.73%	DQB1*05:01:01:03	23	10.36%	40.99%
4	DRB1*15:01:01	15	6.76%	40.99%	DQA1*01:03:01:02	13	5.86%	35.59%	DQB1*02:01:01	17	7.66%	48.65%
5	DRB1*03:01:01:01	12	5.41%	46.40%	DQA1*05:01:01:02	12	5.41%	40.99%	DQB1*03:02:01:01	16	7.21%	55.86%
6	DRB1*11:01:01:01	10	4.50%	50.90%	DQA1*01:02:01:01	10	4.50%	45.50%	DQB1*02:02:01:01	15	6.76%	62.61%
7	DRB1*11:04:01	10	4.50%	55.41%	DQA1*05:05:01:09	10	4.50%	50.00%	DQB1*05:03:01	13	5.86%	68.47%
8	DRB1*14:54:01:02	9	4.05%	59.46%	DQA1*01:01:01:01	9	4.05%	54.05%	DQB1*06:02:01:01	13	5.86%	74.32%
9	DRB1*08:01:01	8	3.60%	63.06%	DQA1*05:05:01:04	9	4.05%	58.11%	DQB1*06:03:01:01	13	5.86%	80.18%
10	DRB1*13:01:01:02	8	3.60%	66.67%	DQA1*01:01:01:03	7	3.15%	61.26%	DQB1*03:03:02:01	7	3.15%	83.33%
11	DRB1*13:01:01:01	7	3.15%	69.82%	DQA1*01:03:01:01	7	3.15%	64.41%	DQB1*04:02:01:01	7	3.15%	86.49%
12	DRB1*15:02:01:01	7	3.15%	72.97%	DQA1*02:01:01:02	7	3.15%	67.57%	DQB1*06:01:01/15	7	3.15%	89.64%
13	DRB1*11:01:01:03	6	2.70%	75.68%	DQA1*01:04:01:01	6	2.70%	70.27%	DQB1*06:04:01	4	1.80%	91.44%
14	DRB1*13:02:01:02	5	2.25%	77.93%	DQA1*05:05:01:01	6	2.70%	72.97%	DQB1*03:01:01:05	3	1.35%	92.79%
15	DRB1*03:01:01:03	4	1.80%	79.73%	DQA1*01:02:01:04	5	2.25%	75.23%	DQB1*05:01:01:05	3	1.35%	94.14%
16	DRB1*04:01:01:03	3	1.35%	81.08%	DQA1*01:04:01:02	5	2.25%	77.48%	DQB1*06:03:01:02	3	1.35%	95.50%
17	DRB1*04:02:01	3	1.35%	82.43%	DQA1*05:01:01:03	5	2.25%	79.73%	DQB1*03:03:02:02	2	0.90%	96.40%
18	DRB1*04:03:01:01	3	1.35%	83.78%	DQA1*01:01:01:02	4	1.80%	81.53%	DQB1*03:01:01:01	1	0.45%	96.85%
19	DRB1*10:01:01:03	3	1.35%	85.14%	DQA1*01:05:01	4	1.80%	83.33%	DQB1*03:01:01:02	1	0.45%	97.30%
20	DRB1*11:03:01	3	1.35%	86.49%	DQA1*04:01:01:03	4	1.80%	85.14%	DQB1*03:01:01:10	1	0.45%	97.75%
21	DRB1*12:01:01/12:10	3	1.35%	87.84%	DQA1*05:05:01:02	4	1.80%	86.94%	DQB1*03:01:01:19	1	0.45%	98.20%
22	DRB1*13:03:01	3	1.35%	89.19%	DQA1*05:05:01:05	4	1.80%	88.74%	DQB1*03:02:01:02	1	0.45%	98.65%
23	DRB1*04:01:01:01	2	0.90%	90.09%	DQA1*01:01:01:05	3	1.35%	90.09%	DQB1*05:01:01:01	1	0.45%	99.10%
24	DRB1*04:05:01:03	2	0.90%	90.99%	DQA1*01:02:01:05	3	1.35%	91.44%	DQB1*05:01:01:02	1	0.45%	99.55%
25	DRB1*04:08:01	2	0.90%	91.89%	DQA1*03:03:01:01	3	1.35%	92.79%	DQB1*05:04	1	0.45%	100.00%
26	DRB1*09:01:02	2	0.90%	92.79%	DQA1*03:02:01:01	2	0.90%	93.69%				
27	DRB1*14:01:01	2	0.90%	93.69%	DQA1*04:02	2	0.90%	94.60%				
28	DRB1*16:02:01:02	2	0.90%	94.60%	DQA1*05:05:01:06	2	0.90%	95.50%				
29	DRB1*01:02:01	1	0.45%	95.05%	DQA1*05:05:01:08	2	0.90%	96.40%				
30	DRB1*03:01:01:02	1	0.45%	95.50%	DQA1*01:01:02	1	0.45%	96.85%				
31	DRB1*04:01:01:02	1	0.45%	95.95%	DQA1*01:02:01:03	1	0.45%	97.30%				
32	DRB1*04:04:01	1	0.45%	96.40%	DQA1*01:03:01:06	1	0.45%	97.75%				
33	DRB1*04:15	1	0.45%	96.85%	DQA1*01:04:01:03	1	0.45%	98.20%				
34	DRB1*08:04:01	1	0.45%	97.30%	DQA1*01:04:01:04	1	0.45%	98.65%				
35	DRB1*10:01:01:01	1	0.45%	97.75%	DQA1*01:10	1	0.45%	99.10%				
36	DRB1*11:01:08	1	0.45%	98.20%	DQA1*04:01:02:01	1	0.45%	99.55%				
37	DRB1*13:05:01	1	0.45%	98.65%	DQA1*05:05:01:03	1	0.45%	100.00%				
38	DRB1*14:05:01:02	1	0.45%	99.10%								
39	DRB1*14:54:01:01	1	0.45%	99.55%								
40	DRB1*16:01:02	1	0.45%	100.00%								

The DQA1 analysis uncovered 37 individual alleles, most common being HLA-DQA1*01:02:02 (15.77%), HLA-DQA1*03:01:01 (7.21%) and HLA-DQA1*02:01:01:01 (6.76%). The highest allelic diversity was observed within DQA1*01 and DQA1*05 allelic families, together accounting for 77.47% of DQA1 allelic variability in our sample population. The most common DQA1*05 representatives were DQA1*05:01:01.02 (5.41%) and DQA1*05:05:01:09 (4.5%) alleles. Two rare alleles of undefined C/WD status were also uncovered, namely, HLA-DQA1*01:10 and HLA-DQA1*04:01:02:01.

Among 25 different HLA-DQB1 alleles observed, the most frequent alleles accounting for 40.99% of DQB1 allelic variability were DQB1*05:02:01:01 (15.77%), DQB1*03:01:01:03 (14.86%) and DQB1*05:01:01:03 (10.36%). These were followed by HLA-DQB1*02:01:01 (7.66%), HLA-DQB1*03:02:01:01 (7.21%) and HLA-DQB1*02:02:01:01 (6.76%) variants. Two most polymorphic allelic families, with at least 5 different allelic specificities in each, were DQB1*05 and DQB1*06 allelic groups. Of interest, HLA-DQB1*03:01:01:19 rare allele of undefined C/WD status was spotted in one donor.

### Haplotype frequencies

A complete list of predicted three and six locus haplotypes is given in Supplementary Tables 6–8. In total, 126 HLA-A~C~B haplotypes were observed in our sample population (Supplementary Table 6), and the list of 20 most commonly linked allelic combinations (>1%) is available in Table 3. Only one HLA-A~C~B haplotype, the ancestral European combination A*01:01:01:01~C*07:01:01~B*08:01:01, was present in more than 5% of tested individuals. The second, third and fourth most frequent class I haplotypes were A*11:01:01:01~C*04:01:01:01~B*35:01:01 (3.6%) A*03:01:01:01~C*07:02:01~ B*07:02:01 (3.11%) and A*02:01:01~C*06:02:01:01~B*57:01:01:01 (2.7%), respectively. The remaining three-loci class I haplotypes were observed ≤5 times in our data set.

**Table 3:** The most frequent (>1%) HLA~A~B~C haplotypes in East Croatia blood donor volunteers (n = 111).

	HLA-A~B~C			Observed (n)	HF	cF
1.	A*01:01:01:01	B*08:01:01	C*07:01:01	15.00	6.76%	6.76%
2.	A*11:01:01:01	B*35:01:01	C*04:01:01:01	8.00	3.60%	10.36%
3.	A*03:01:01:01	B*07:02:01	C*07:02:01	6.89	3.11%	13.47%
4.	A*02:01:01	B*57:01:01:01	C*06:02:01:01	6.00	2.70%	16.17%
5.	A*02:01:01	B*27:02:01:01	C*02:02:02	5.00	2.25%	18.42%
6.	A*02:01:01	B*35:01:01	C*04:01:01:06	5.00	2.25%	20.67%
7.	A*02:01:01	B*35:03:01	C*04:01:01:01	5.00	2.25%	22.93%
8.	A*02:01:01	B*44:02:01:01	C*05:01:01	5.00	2.25%	25.18%
9.	A*03:01:01:01	B*35:03:01	C*04:01:01:01	5.00	2.25%	27.43%
10.	A*02:01:01	B*44:27:01	C*07:04:01	4.00	1.80%	29.23%
11.	A*11:01:01:01	B*52:01:01	C*12:02:02	4.00	1.80%	31.03%
12.	A*02:01:01	B*27:05:02	C*02:02:02	3.89	1.75%	32.79%
13.	A*02:01:01	B*18:01:01	C*07:01:01	3.00	1.35%	34.14%
14.	A*02:01:01	B*52:01:01	C*12:02:02	3.00	1.35%	35.49%
15.	A*03:01:01:01	B*15:01:01:01	C*07:04:01	3.00	1.35%	36.84%
16.	A*23:01:01:01	B*44:03:01	C*04:01:01:01	3.00	1.35%	38.19%
17.	A*24:02:01:01	B*13:02:01:01	C*06:02:01:01	3.00	1.35%	39.54%
18.	A*24:02:01:01	B*35:02:01	C*04:01:01:06	3.00	1.35%	40.90%
19.	A*24:02:01:01	B*51:01:01	C*15:02:01:01	3.00	1.35%	42.25%
20.	A*02:01:01	B*07:02:01	C*07:02:01	2.88	1.30%	43.54%

The HLA-DRB1~DQA1~DQB1 haplotype diversity is presented in Supplementary Table 7 and those observed ≥3 times are enlisted in Table 4. Among 75 different haplotypes, DRB1*16:01:01~DQA1*01:02:02~DQB1*05:02:01:01 (13.51%) was the most frequent in our population. The next high-ranking haplotypes, were DRB1*07:01:01~ DQA1*02:01:01:01~DQB1*02:02:01:01 (5.32%), DRB1*03:01:01:01~ DQA1*05:01:01:02~DQB1*02:01:01 (4.96%), DRB1*15:01:01~DQA1*01:02:01:01~ DQB1*06:02:01:01 (4.50%) and DRB1*01:01:01~DQA1*01:01:01:01~ DQB1*05:01:01:03 (4.05%).

**Table 4:** The most frequent (>1%) HLA-DQA1~DQB1~DRB1 haplotypes in East Croatia blood donor volunteers (n = 111).

No.	HLA-DQA1~DQB1~DRB1			Observed (n)	HF	cF
1.	DQA1*01:02:02	DQB1*05:02:01:01	DRB1*16:01:01	30.00	13.51%	13.51%
2.	DQA1*02:01:01:01	DQB1*02:02:01:01	DRB1*07:01:01	11.82	5.32%	18.84%
3.	DQA1*05:01:01:02	DQB1*02:01:01	DRB1*03:01:01:01	11.00	4.96%	23.79%
4.	DQA1*01:02:01:01	DQB1*06:02:01:01	DRB1*15:01:01	10.00	4.50%	28.30%
5.	DQA1*01:01:01:01	DQB1*05:01:01:03	DRB1*01:01:01	9.00	4.05%	32.35%
6.	DQA1*01:01:01:03	DQB1*05:01:01:03	DRB1*01:01:01	7.00	3.15%	35.50%
7.	DQA1*01:03:01:01	DQB1*06:01:01/15	DRB1*15:02:01:01	7.00	3.15%	38.66%
8.	DQA1*01:03:01:02	DQB1*06:03:01:01	DRB1*13:01:01:01	7.00	3.15%	41.81%
9.	DQA1*01:04:01:01	DQB1*05:03:01	DRB1*14:54:01:02	6.00	2.70%	44.51%
10.	DQA1*05:05:01:09	DQB1*03:01:01:03	DRB1*11:04:01	5.00	2.25%	46.76%
11.	DQA1*01:01:01:02	DQB1*05:01:01:03	DRB1*01:01:01	4.00	1.80%	48.57%
12.	DQA1*01:02:01:04	DQB1*06:04:01	DRB1*13:02:01:02	4.00	1.80%	50.37%
13.	DQA1*01:03:01:02	DQB1*06:03:01:01	DRB1*13:01:01:02	4.00	1.80%	52.17%
14.	DQA1*04:01:01:03	DQB1*04:02:01:01	DRB1*08:01:01	4.00	1.80%	53.97%
15.	DQA1*05:01:01:03	DQB1*02:01:01	DRB1*03:01:01:03	4.00	1.80%	55.77%
16.	DQA1*02:01:01:02	DQB1*03:03:02:01	DRB1*07:01:01	3.82	1.72%	57.49%
17.	DQA1*02:01:01:01	DQB1*03:03:02:01	DRB1*07:01:01	3.18	1.43%	58.93%
18.	DQA1*02:01:01:02	DQB1*02:02:01:01	DRB1*07:01:01	3.18	1.43%	60.36%
19.	DQA1*01:01:01:05	DQB1*05:01:01:03	DRB1*01:01:01	3.00	1.35%	61.71%
20.	DQA1*01:02:01:05	DQB1*06:02:01:01	DRB1*15:01:01	3.00	1.35%	63.06%
21.	DQA1*03:01:01	DQB1*03:02:01:01	DRB1*04:01:01:03	3.00	1.35%	64.41%
22.	DQA1*03:01:01	DQB1*03:02:01:01	DRB1*04:02:01	3.00	1.35%	65.77%
23.	DQA1*03:01:01	DQB1*03:02:01:01	DRB1*04:03:01:01	3.00	1.35%	67.12%
24.	DQA1*05:05:01:02	DQB1*03:01:01:03	DRB1*11:01:01:01	3.00	1.35%	68.47%
25.	DQA1*05:05:01:04	DQB1*03:01:01:03	DRB1*11:01:01:01	3.00	1.35%	69.82%
26.	DQA1*05:05:01:05	DQB1*03:01:01:03	DRB1*13:03:01	3.00	1.35%	71.17%
27.	DQA1*05:05:01:09	DQB1*03:01:01:03	DRB1*11:01:01:03	3.00	1.35%	72.52%

Of interest, rare DRB1*01:01:01~DQA1*01:02:01:03~DQB1*05:04, and two tentative DRB1*13:01:01:02~DQA1*01:10~DQB1*06:03:01:01 and DRB1*04:05:01:03~ DQA1*05:05:01:06~DQB1*03:01:01:03 haplotypes (Supplementary Table 8) with no previous entries within the Allele Frequency database so far, were spotted once.

Among 181 six-loci haplotypes (Supplementary Table 8), 10 appeared in three or more copies (Table 5), and the most frequent was the ancestral haplotype A*01:01:01:01~C*07:01:01~B*08:01:01~DRB1*03:01:01:01~DQA1*05:01:01:02~DQB1*02:01:01 (3.6%). Three six-loci combinations (A*02:01:01~C*07:01:01~B*18:01:01~ DRB1*11:04:01~DQA1*05:05:01:09~DQB1*03:01:01:03, A*03:01:01:01~C*07:02:01~ B*07:02:01~DRB1*16:01:01~DQA1*01:02:02~DQB1*05:02:01:01, and A*11:01:01:01~ C*04:01:01:01~B*35:01:01~DRB1*16:01:01~DQA1*01:02:02~DQB1*05:02:01:01), which have already been reported in Italian population^27^, Turkish minority in Germany^20^, and Croatian families^18^, were also commonly (1.8%) observed. Haplotypes characteristic for South European populations of Slavic background or admixture were also found^17^, namely A*02:01:01~C*02:02:02~B*27:02:01:01~DRB1*16:01:02~ DQA1*01:02:02~DQB1*05:02:01:01 (1.8%), and even more unique A*02:01:01~C*02:02:02~B*27:05:02~DRB1*01:01:01~DQA1*01:01:01:03~DQB1*05:02:01:01 (0.45%), A*02:01:01~C*07:02:01~B*27:05:02~DRB1*01:01:01~ DQA1*01:01:01:01~DQB1*05:01:01:03 (0.45%) and A*02:01:01~C*07:02:01~ B*27:05:02~DRB1*01:01:01~ DQA1*01:01:01:03~ DQB1*03:01:01:03 (0.45%). Nevertheless, a limited power in identifying low-frequency haplotypes should be noticed in our sample, where majority of haplotypic combinations were observed only once, and most frequent variants cover only 17.57% of our population haplotypic diversity.

**Table 5:** HLA-A~B~C~DRB1~DQA1~DQB1 extended haplotypes with estimated haplotype (HF) and cumulative frequency (cF) of 1% or more in East Croatian blood donor volunteers (n = 111).

No.	HLA-A~B~C~DQA1~DQB1~DRB1						n	HF	cF
**1**	A*01:01:01:01	B*08:01:01	C*07:01:01	DQA1*05:01:01:02	DQB1*02:01:01	DRB1*03:01:01:01	8.00	3.60%	3.60%
**2**	A*02:01:01	B*18:01:01	C*07:01:01	DQA1*05:05:01:09	DQB1*03:01:01:03	DRB1*11:04:01	4.00	1.80%	5.41%
**3**	A*02:01:01	B*27:02:01:01	C*02:02:02	DQA1*01:02:02	DQB1*05:02:01:01	DRB1*16:01:01	4.00	1.80%	7.21%
**4**	A*03:01:01:01	B*07:02:01	C*07:02:01	DQA1*01:02:02	DQB1*05:02:01:01	DRB1*16:01:01	4.00	1.80%	9.01%
**5**	A*11:01:01:01	B*35:01:01	C*04:01:01:01	DQA1*01:02:02	DQB1*05:02:01:01	DRB1*16:01:01	4.00	1.80%	10.81%
**6**	A*02:01:01	B*13:02:01:01	C*06:02:01:01	DQA1*02:01:01:01	DQB1*02:02:01:01	DRB1*07:01:01	3.00	1.35%	12.16%
**7**	A*02:01:01	B*44:27:01	C*07:04:01	DQA1*01:02:02	DQB1*05:02:01:01	DRB1*16:01:01	3.00	1.35%	13.51%
**8**	A*02:01:01	B*52:01:01	C*12:02:02	DQA1*01:03:01:01	DQB1*06:01:01/15	DRB1*15:02:01:01	3.00	1.35%	14.87%
**9**	A*11:01:01:01	B*35:01:01	C*04:01:01:01	DQA1*01:01:01:01	DQB1*05:01:01:03	DRB1*01:01:01	3.00	1.35%	16.22%
**10**	A*24:02:01:01	B*13:02:01:01	C*06:02:01:01	DQA1*02:01:01:01	DQB1*02:02:01:01	DRB1*07:01:01	3.00	1.35%	17.57%

## Discussion

This study represents the first report of HLA diversity in an east Croatian population of healthy blood donors, as studied by the next generation sequencing of 6 HLA genes, providing extensive exon/intron coverage with minimum ambiguity. For the first time, the allele frequencies and the extended six gene haplotypes of Croats were examined at the 4-field resolution level and compared to the largest repository of HLA class I and class II data in Croatia, the Croatian bone marrow donor registry (CBMDR).

The comparison of HLA-A allele frequencies between our and CBMDR inventory did not reveal significant differences, and the ranking hierarchy of the most common A*02:01:01, A*01:01:01:01, A*03:01:01:01, and A*24:01:01:01 alleles was also the same. Greater differences in frequency rate between general and east Croatian population were, however, noticed among HLA-B allelic variants. The HLA-B*51:01 allelic group was the most frequent in both general and east Croatian cohort, but the frequency rank of the remaining HLA-B allelic variants was different, which was particularly evident for our 6^th^ ranking B*18:01 allele group, previously reported as the 2^nd^ most frequent allelic variant in the CBMDR (8.16%)^17^ and one Croatian family study (8.27%)^18^. Among five different B*18 alleles detected in the Croats so far^28^, we observed only two, the B*18:01:01 (5.14%) and the B*18:03 (0.90%), resulting in a B*18:01 distribution closer to those reported in Armenians^29^, Germans^30^, Austrian and the Turkish minority in Germany^20^, Bulgarian Roma individuals^31^, and Iranian Kurds^32^. Moreover, the frequency of the 2^nd^ most common HLA-B allelic group in our cohort, the HLA-B*35:01 (8.11%, ranked 5^th^ in the CBMDR), was more similar to those observed in Turks^20^, Serbians^9^, and Italians^27^. A strong influence of south-eastern European populations on the HLA makeup of eastern Croats was further supported by the high prevalence of the HLA-B*27:02:01:01 variant, which fits well into the B*27:02 frequency gradient diminishing from the Middle East towards the Central and West European countries. In support, the observed B*27:02:01:01 frequency (3.6%) seems to be in close agreement with the B*27:02 cline extending across the south-eastern Tunisian (5.8%)^33^, Bulgarian (4.6%)^34^, CBMDR (2.14%)^17^, Czech (1.9%)^9^, and Polish (1.5%)^35^ populations. The B*44:27:01 allele, an east European marker considered a rare variant according to the “Rare Alleles Detector” tool^9^, was also noticed in our cohort at a relatively high frequency (2.7%), contrasting observations from Croatian HSCT patients (1.18%)^19^, Czech National Marrow Donors Registry (0.69%)^9^, as well as Polish (0.8%)^35^, English (0.19%)^36^, and Argentinian blood donors (0.07%)^37^. As minor allele within the functionally identical B*44:02 G group, the B*44:27 variant has been reported at a relative ratio frequency of >5% among the B*44:02:01G-positive Bulgarian (36.82%), Hungarian (9.4%), Slovenian (25.60%) and Portuguese (6.17%) individuals^38^. In our sample, the B*44:27:01 relative ratio frequency within the B*44:02:01G-positive individuals (37.5%) sets the local estimate at the upper boundary.

Several ranking differences were detected between the general Croatian and our population at the HLA-C loci as well. The 1^st^ (C*07:01, AF = 21.77%), and the 2^nd^ (C*04:01, AF = 15.59%) highest ranking alleles from the CBMDR inventory were ranked 2^nd^ (14.41%) and 1^st^ (18.01%) in east Croatian blood donors, respectively. The ranking sequence in our cohort continued with C*02:02:02 (9.46%), and C*07:02:01 (8.11%) allele group, which have been deemed 4^th^ and 5^th^ highest ranking alleles in the CBMRD. Overall, the ranking hierarchy of HLA-C allele groups in our sample was more similar to the one reported for Greece, and the Turkish minority in Germany^20^.

Compared to class I, class II loci exhibited a higher level of heterozygosity at the 4-field level (molecular variation in introns and UTR regions), allowing us to see three or more different allelic variants within the particular 3-field G allelic group. For instance, the highest allelic variability was found within the DQA1*05:05:01 G (8 different alleles), DQA1*01:01:01 G (5), DQA1*01:02:01 G (5), DQA1*01:04:01 G (5), DQB1*03:01:01 G (6) and DQB1*05:01:01 G (4), whereas up to 3 allelic variants were revealed within the DRB1*03:01:01 G, DRB1*04:01:01 G, DRB1*11:01:01 G and DQA1*03:01:01 G group^6,7^.

Compared to Croatian general population and neighbouring nations, the frequency and order of DRB1*16:01 (13.96%) and DRB1*03:01 (7.66%), the 1^st^ and 5^th^ most common DRB1 allele groups in our cohort, were opposite to CBMDR allelic hierarchy (9.41% (4^th^) and 10.01% (1^st^), respectively), but similar to East European (Bulgaria 15.5%, 8.18%, respectively)^34^, and Mediterranean populations (Macedonia 14.9%, 6.7% and Greece 11.5%, 6.5%, respectively)^39–41^. At the same time, the frequency ratio of DRB1*11:01:01:01 and DRB1*11:04:01 alleles in our sample was in concordance with the CBMDR data, confirming equal frequency rate of these two alleles across Croatia, in contrast to higher DRB1*11:01 prevalence in the north, and DRB1*11:04 in the south of Europe^17^. In line with the high prevalence and LD patterns of DRB1*16:01:01, DQA1*01:02:02 (15.77%) and DQB1*05:02:01:01 (15.77%) alleles were the most commonly observed DQA1 and DQB1 variants at the 4-field level among east Croatian blood donors. However, looking at the 2-field level, DQA1*01:02 (24.32%), DQA1*05:05 (17.1%) and DQB1*03:01 (18.01%) allelic families were found at a higher frequency, emphasizing significant allelic heterogeneity within the particular allelic group. The most striking observation was a high frequency of the DQA1*05:05 allele (17.1%), a common South-European allelic variant (EFI CWD catalogue v.1.0.) that has been reported at much lower frequency only in the autochthonous Croatian population from the Gorski Kotar (2.4%)^24^, but not in the CBMDR database. Thus far, the DQA1*05:05 allele has been reported for a handful of European populations^9^, and the frequency observed in our cohort is currently surpassed only by the prevalence observed among Czechs (20.6%)^42^, north Italians (30.5%)^9^ and Greeks (32.5%)^43^. Additional differences at the DQB1 locus were noticed after comparing DQB1*05:02 and DQB1*02:01 frequencies, with DQB1*05:02 being more prevalent in eastern (east vs. general CRO; 15.77% vs. 8.53%) and DQB1*02:01 in general Croatian population (east vs. general CRO; 7.66 vs. 12.94%), partly reflecting a positive, North-West to South-East gradient of DQB1*05:02 frequencies. In contrast, an inverse distribution was observed for DQB1*02:01 across Europe, with the highest DQB1*05:02 and DQB1*02:01 prevalence observed in Greeks (19.8%)^44^ and Englishmen (33%)^45^, respectively.

The haplotype distribution in our cohort is in close agreement with previously reported class I and class II associations in CBMDR and Croatian families, with few interesting differences found in our population of eastern Croats. Next to the pan-European (A*01:01:01:01~C*07:01:01~B*08:01:01~DRB1*03:01:01:01~DQA1*05:01:01:02~DQB1*02:01:01) and Mediterranean (A*02:01:01~C*07:01:01~B*18:01:01~ DRB1*11:04:01~DQA1*05:05:01:09~DQB1*03:01:01:03) haplotypes, two notable exceptions were extended A*03:01~C*07:02~B*07:02~DRB1*16:01 and A*11:01~B*35:01~C*04:01~DRB1*16:01 variants, both commonly observed in Macedonian families^46^, as well as Austrian, Croatian, Bosnian-Herzegovinian, Italian, Romanian, Greek and Turkish minority in Germany^20^, but not in the CBMDR^17^ or Croatian family study^18^ (Table 5), where A*03:01~B*07:02 was more frequently found with DRB1*15:01, and A*11:01~B*35:01 with DRB1*01:01. Moreover, DRB1*11:01, -*11:04, -*12:01 and -*13:03 alleles were more frequently associated with DQA1*05:01~DQB1*03:01 haplotype in general, but not east Croatian population, where above-mentioned DRB1 alleles were in strong linkage disequilibrium with DQA1*05:05~DQB1*03:01 combination (Supplementary Table 8). Similar linkage patterns were, however, noticed by comparing extended A*02:01~B*27:02~C*02:02~ DRB1*16:01~DQA1*01:02~DQB1*05:02 (1.8%) and A*02:01~B*27:05~C*02:02~ DRB1*01:01~DQA1*01:01~DQB1*05:02 (0.45%) haplotypes with the CBMDR and Southeast European haplotype inventory available from the German Bone Marrow Donor Registry (DKMS)^20^. Compared to our database, the A*02:01~B*27:02~C*02:02~DRB1*16:01 haplotype was more frequent only in Bulgarians (2.73%)^34^, but more similar to CBMDR (0.74%)^17^, Polish (1%), Bosnian and Herzegovinian (0.9%), and Croatian (0.834%) minority in Germany^20^.

The A*02:01~B*27:05~C*02:02~DRB1*01:01, was observed at lower frequency in Polish (0.27%), Austrian (0.12%), Bosnian and Herzegovinian (0.065%), Greek (0.026%) and Romanian (0.067%) minority in DKMS^20^, compared to CBMDR (0.85%) and our cohort (0.45%), possibly supporting local origin of this haplotype. Moreover, it was interesting to see a relatively high prevalence of the extended A*02:01~B*44:27~C*07:04~DQA1*01:02~DQB1*05:02~DRB1*16:01 (1.35%) haplotype, for whom there is no population data in Allele Frequency Net database. Recent study performed in B*44-positive Croatian subjects reported a very strong and almost exclusive linkage disequilibrium between B*44:27 and C*07:04 alleles, whereas B*44:02, -*44:03 and -*44:05 were more commonly seen in association with C*05:01, -*04:01 and -*02:02, respectively^47^. This is in complete agreement with the B*44 extended haplotypes found in our cohort [A*02:01:01~B*44:02:01:01~C*05:01:01~DRB1*13:01:01:01 (0.9%), A*23:01:01:01~B*44:03:01~C*04:01:01:01~DRB1*07:01:01 (1.35%), and A*02:01:01~B*44:05:01~C*02:02:02~DRB1*01:01:01 (0.45%)]. Our data further coincided with the DKMS reports on the A*24:02~C*15:02~B*51:01~ DRB1*16:01~DQB1*05:02 and A*24:02~C*06:02~B*13:02 ~DRB1*07:01~DQB1*02:02 haplotype frequency in Croatian, Greece, Bosnian and Herzegovinian and Polish minority in Germany^20^. In addition, several extended variants of common Mediterranean and Southeastern European haplotypes were also observed in our population; but at a lower frequency; namely the A*02:01:01:01~C*12:03:01~B*51:01:01~DRB1*11:01:01:01 ~DQB1*03:01:01:03 (0.45%) and A*24:02:01:01~C*04:01:01:06~B*35:02:01~ DRB1*11:04:01~DQB1*03:01:01:02 (0.45%), which were most frequent in Greece^41^, Albania^48^, Italian and Turkish minority in DKMS^20^ (reduced haplotype variant); and the A*02:01:01~C*02:02:01~B*51:01:01~DRB1*13:01:01:01~DQB1*03:01:01:19 (0.45%) variant, most frequently found in Bulgarians (reduced haplotype variant)^34^. Significant influence of Central and Western European countries on the east Croatian HLA profile is nonetheless also evident through higher prevalence of two extended, common European haplotypes, the A*11:01:01:01~C*04:01:01:01~B*35:01:01~DRB1*01:01:01~ DQA1*01:01:01:01~DQB1*05:01:01:03 (1,35%) and A*02:01:01~C*06:02:01:01~ B*13:02:01:01~DRB1*07:01:01~DQA1*02:01:01:01~DQB1*02:02:01:01 (1.35%), which occur at similar frequency in Swedish^9^, Polish^35^, CBMDR^17^, Austrian, Italian and the Portuguese minority population from the DKMS inventory^20^.

In conclusion, the present study provides an in-depth characterisation of HLA diversity in eastern Croats, revealing distinctive allele and haplotype detail consistent with the complex population history of the studied geographic region. The data complement and refine the existing estimates of HLA diversity in the Croatian population, increase population and geographic coverage by NGS data, and add granularity to clinically and genetically relevant HLA data. The study represents a useful reference for population and HLA-disease association studies; however, larger sample size and sequence coverage, particularly for the DQB1 and DRB1 genes, remain a prerequisite for the future studies.

## Materials and Methods

### Subjects

The study collection consisted of 120 healthy, unrelated, blood donor volunteers (34 female, 86 male, 20–61 years of age, median age 36 years) originating from five eastern Croatia counties; Osijek-Baranja county (n = 80), Vukovar-Srijem county (n = 22), Brod-Posavina county (n = 9) Požega-Slavonia county (n = 4) and Virovitica-Podravina county (n = 5). All participants were recruited during voluntary blood donations in county Red Cross branches or at the Clinical Institute of Transfusion Medicine, Osijek University Hospital. Prior to the blood sampling, completed health questionnaire forms were collected from all donors, to select individuals with no personal of family history of various autoimmune and cardiovascular diseases, stroke or carcinoma. Informed consent in written form was collected from all subjects. All investigations were conducted in accordance with the 1964 Declaration of Helsinki and subsequent legal instruments. Ethical approval was provided by the University Hospital Osijek Ethics Committtee (No. 25–1:831–3/2015).

### DNA extraction and quantification

Genomic DNA was extracted from 200 μl peripheral blood samples mixed with EDTA, using High Pure PCR Template Preparation Kit (Roche Diagnostics, Mannheim, Germany) according to the instructions in the manufacturer leaflet. Quantity and quality of isolated genetic material was verified by OD_260_/OD_280_ > 1.8 and OD_260_/OD_230_ > 1.6 measurements performed on IMPLEN NanoPhotometer P-Class P-330 (IMPLEN GmbH, Munich, Germany).

### Long-range PCR amplification, pooling and clean-up of PCR products

HLA genotypes for HLA-A, -B, -C, -DRB1, -DQA1, and -DQB1 loci were determined using high-resolution Omixon Holotype HLA 96/7 and 24/7 (Omixon Biocomputing Ltd, Budapest, Hungary) configuration kits on Illumina MiSeq next-generation sequencing platform. The Omixon Holotype PCR primers allowed amplification of the entire HLA-A (5′UTR nucleotide position: −78; 3′UTR nucleotide position: 3081), -B (5′UTR: −35; 3′UTR: 2680), -C (5′UTR: −122; 3′UTR: 2915), and -DQA1 (5′UTR: −281; 3′UTR: 5750) loci. Class II sequence analysis evaluated nucleotides from intron 1 to 3′ UTR of HLA-DQB1 (intron 1: 645; 3′UTR: 6469), whereas –DRB1 locus was sequenced from intron 1 (nucleotide position 4753) to intron 4 (nucleotide position 9135) (Fig. 2). The HLA genotyping workflow was initiated by long-range PCR amplification of class I and class II HLA loci in a separate, sample and locus specific 25 µl reactions, comprising 2.5 µl of PCR buffer, 1.25 µl of dNTP mix, 2 µl of locus specific primers, 0.4 µl of LR PCR enzyme, and 5 µl of genomic DNA (≈30 ng/µl). Combined DQB1 enhancer (5.6 µl/sample) was added to the DQB1 master mix only. The conditions for class I gene amplification on Mastercycler nexus thermal cycler (Eppendorf, Hamburg, Germany) were set as follows: 95 °C for 3 min, followed by 35 cycles of 95 °C for 15 s, 65 °C for 30 s and 68 °C for 5 min, and a final incubation at 68 °C for 10 min. For class II genes, the conditions were: 95 °C for 3 min, 35 cycles of 93 °C for 15 s, 60 °C for 30 s and 68 °C for 9 min, followed by final extension at 68 °C for 10 min. Amplicon size was validated by 2% agarose gel electrophoresis and DNA quantitated on EnSpire Multimode plate reader (PerkinElmer, Waltham, MA, USA) using QuantiFluor fluorescent dsDNA staining system (Promega, Madison, Wisconsin, USA). All six amplicons from one individual sample were pooled into a final 35 µl volume on a fresh 96-well PCR plate, and purified from residual primers and unincorporated nucleotides with the use of ExoSAP-iT enzyme mix (Affymetrix Inc., Santa Clara, CA, USA).

### Library construction, normalisation and sequencing on MiSeq

Library preparation, in the next few steps, included fragmentation of each six-locus amplicon pool, fragment end repair and ligation with sample-specific indexed adaptors. Equal aliquots of indexed sample-specific libraries were subsequently combined into a 900 µl pooled library volume and mixed with 900 µl of the AMPure XP beads (Beckman Coulter, Beverly, Massachusetts, USA) to carry out magnetic bead-based library cleanup. Pooled library fragments ranging between 650 and 1300 bps in size were subsequently selected on Pippin Prep instrument (Sage Science, Beverly, Massachusetts, USA). The concentration of the size selected library was determined on LightCycler 480 II (Roche Diagnostics, Mannheim, Germany) real-time PCR instrument using KAPA Sybr Fast qPCR Master Mix (KAPA Biosystems, Boston, Massachusetts, USA) and DNA standards ranging from 0.02 pM to 20 pM concentrations. Prior to sequencing, library was diluted to a 2 nM concentration, loaded on a MiSeq flow cell (Illumina, San Diego, CA, USA) and sequenced in a single 500 cycle (V2) paired-end sequencing run. Collected reads were exported in fastq format and analysed with the Omixon Twin software v3.0.0. and the IPD-IMGT/HLA database Release 3.30.0_5 (November 2017).

### Data analysis

The best matching alleles were selected according to the alignment statistics (described in section 4.6), and homology to alleles available in the IMGT/HLA 3.30.0_5 database^6,7^. If more than one allele call was available for a specific locus, the ambiguity was resolved by re-analysis of increased number of reads processed from the input files. The remaining ambiguous allele calls (presented in Supplementary Table 1) were referenced against the “Omixon Holotype HLA and Omixon HLA Twin known product limitations” (missing data on SNPs or INDEL variations within the unsequenced 3′ UTR, 5′ UTR and intron 1/exon 1 regions), and were hence reported as ambiguous (i.e. DQB1*06:01:01/15) or up to the third field level only (i.e. DQB1*05:03:01). The Common and Well-Documented (CWD) allele catalogue (version 2.0.), and “Rare Allele Detector” tool (www.allelefrequencies.net), were used for the identification of rare HLA alleles. Nine out of 120 samples were excluded from this study due to Omixon Twin quality control failure. HLA-A, -B, -C, DRB1, -DQA1, -and -DQB1 loci were successfully sequenced in all remaining samples (n = 111).

### Quality control (QC) metrics

The Omixon Twin software combines statistical alignment and *de novo* assembly algorithms for robust allele calling. The default minimum number of reads required for reliable locus mapping was set at ≥2500 for class I, and ≥5000 for class II loci. A read length of 200 bp or greater was a prerequisite for passing QC criteria, and together with additional quality metrics (read quality, noise ratio, consensus phasing, allele imbalance, crossmapping reads, mismatch count) assured the accuracy and confidence of allele assignments. The minimum exon/intron coverage threshold supporting the consensus sequence at the weakest position was set at ≥30 reads.

### Statistics

Allelic frequencies were determined by direct counting. Arlequin version 3.5.2.2^49^ was used to calculate expected and observed heterozygosity, exact deviations from Hardy-Weinberg equilibrium (a modified version of the Markov-chain random walk algorithm described by Guo and Thomson, 10^6^ steps in Markov chain, 10^5^ dememorization steps)^50^, and maximum-likelihood haplotype frequencies (an iterative Expectation-Maximization algorithm, convergence criterion ε = 10^−7^, maximum number of iterations = 1000, 50 random initial conditions)^51^. A series of linkage disequilibrium (LD) measures (D'^52,53^, Wn^54^) was provided for each pair of loci by using the Pypop 0.7.0 software^55^. The empirical P-values were obtained by permutation testing (1000 randomizations).

## Supplementary information

Supplementary Information.
