# Supplementary material for: HLA-A, -B, -C, -DRB1, -DQA1, and -DQB1 allele and haplotype frequencies defined by next generation sequencing in a population of East Croatia blood donors
Source: Sci Rep. 2020 Mar 26;10:5513. doi: 10.1038/s41598-020-62175-9 (PMC7099076; doi:10.1038/s41598-020-62175-9)
Supplement: Supplementary file 1 — Supplementary Information. [file 41598_2020_62175_MOESM1_ESM.doc]

**Supplementary information**

HLA-A, -B, -C, -DRB1, -DQA1, and -DQB1 allele and haplotype frequencies defined by next generation sequencing in a population of East Croatia blood donors

Stana Tokić ^1^ *, Veronika Žižkova ^4^, Mario Štefanić ^2^*, Ljubica Glavaš-Obrovac ^1^, Saška Marczi ^3^, Marina Samardžija ^3^, Katerina Sikorova ^4^ and Martin Petrek ^4^ *

^1^ Dept. of Medical Chemistry, Biochemistry and Clinical Chemistry, Faculty of Medicine, University of Osijek, J. Huttlera 4, HR-31000 Osijek, Croatia

^2^ Dept. of Nuclear Medicine and Oncology, Faculty of Medicine, University of Osijek, J. Huttlera 4, HR-31000 Osijek, Croatia

^3^ Dept. of Laboratory Diagnostics and Clinical Transfusion Medicine, Clinical Institute of Transfusion Medicine, Osijek University Hospital, J. Huttlera 4, HR-31000 Osijek, Croatia

^4^ Dept. of Pathological Physiology, Faculty of Medicine and Dentistry, Palacký University, Hnevotinska 3 775 15 Olomouc, Czech Republic

***** Correspondence: stokic@mefos.hr; mstefanic@mefos.hr; Martin.Petrek@fnol.cz

**Supplementary Table 1. Characterization of number and position of SNVs among ambiguos alleles at each HLA locus**

| **Third/fourth field ambiguities and expression-related ambiguities (null/low expressed allele)** | | | | |
| --- | --- | --- | --- | --- |
| **G group (No. observed)** | **Lowest numbered allele in G group** | **Exon identical allele** | **Position of SNV causing the ambiguity (IMGT position)** | **Shared with other alleles** |
| **HLA-A*02:01:01 (72)** | HLA-A*02:01:01:01 | HLA-A*02:01:01:02L | SNP T/C (5'UTR, pos -101) | No |
|  |  | HLA-A*02:01:01:16 | SNP C/T (3'UTR, pos 3082) | No |
| **HLA-B*07:02:01 (15)** | HLA-B*07:02:01:03 | HLA-B*07:02.01:01 | SNP C/T (3'UTR, pos 3358) | No |
| **HLA-B*07:05:01 (1)** | HLA-B*07:05:01:01 | HLA-B*07:05:01:02 | SNP T/C (3'UTR, pos 3740) SNP T/C (3'UTR, pos 3768) | No No |
| **HLA-B*08:01:01 (18)** | HLA-B*08:01:01:01 | HLA-B*08:01:01:02 | SNP T/C (3'UTR, pos 2803) | No |
| **HLA-B*18:01:01 (12)** | HLA-B*18:01:01:02 | HLA-B*18:01:01:04 | SNP C/T (5'UTR, pos -93) | No |
|  |  | HLA-B*18:01:01:05 | SNP G/A (3'UTR, pos 3725) SNP A/G (3'UTR, pos 3752) | Yes (B*40:01:02:04) Yes (B*40:02:01:02) |
| **HLA-B*27:05:02 (9)** | HLA-B*27:05:02:01 | HLA-B*27:05:02:05 | SNP C/T (3'UTR, pos 3609) | Yes (B*40:01:02:01) |
| **HLA-B*35:01:01 (18)** | HLA-B*35:01:01:01 | HLA-B*35:01:01:02/04/05/06 | SNP G/A (3'UTR, pos 2932) | Yes |
| **HLA-B*35:02:01 (4)** | HLA-B*35:02:01:01 | HLA-B*35:02:01:02 | SNP A/C (3'UTR, pos 3184) SNP T/G (3'UTR, pos 3251) SNP A/G (3'UTR, pos 3255) | No |
| **HLA-B*35:03:01 (15)** | HLA-B*35:03:01:01 | HLA-B*35:03:01:03 | INS TGGCAGGTCTG (3'UTR, pos 2711) | No |
|  |  |  | SNP C/T (3'UTR, pos 2707) | No |
| **HLA-B*39:01:01 (4)** | HLA-B*39:01:01:02L | HLA-B*39:01:01:03 | DEL TC (5'UTR, pos -151) | Yes (B*39:01:01:05) |
|  | HLA-B*39:01:01:03 | HLA-B*39:01:01:05 | SNP G/A (3'UTR, pos 3696) | No |
| **HLA-B*40:01:02 (3)** | HLA-B*40:01:02:01 | HLA-B*40:01:02:02 | SNP A/G (5'UTR, pos -216) | No |
|  |  | HLA-B*40:01:02:04 | SNP A/G (3'UTR, pos 3725) | Yes (B*18:01:01:02) |
|  |  | HLA-B*40:01:02:06 | SNP C/T (3'UTR, pos 3511) SNP A/G (3'UTR, pos 3529) SNP T/C (3'UTR, pos 3609) SNP A/C (3'UTR, pos 3668) | Yes (B*51:01:01:01) No Yes (B*51:01:01:01) No |
| **HLA-B*40:02:01 (3)** | HLA-B*40:02:01:01 | HLA-B*40:02:01:02 | SNP T/A (3'UTR, pos 3687) SNP A/G (3'UTR, pos 3752) | No Yes (B*51:01:01:05) |
|  |  | HLA-B*40:02:01:03 | SNP T/A (3'UTR, pos 3507) | Yes (B*44:03:01:10; B*51:01:01:01) |
| **HLA-B*44:03:01 (5)** | HLA-B*44:03:01:01 | HLA-B*44:03:01:04 | SNP A/G (3'UTR, pos 2723) | No |
|  |  | HLA-B*44:03:01:10 | SNP G/T (3'UTR, pos 3412) SNP C/T (3'UTR, pos 3472) SNP A/T (3'UTR, pos 3507) SNP C/T (3'UTR, pos 3572) | No No Yes (B*40:02:01:03; B*51:01:01:05) No |
| **HLA-B*49:01:01 (2)** | HLA-B*49:01:01:01 | HLA-B*49:01:01:02 | SNP T/C (3'UTR, pos 3020) SNP T/C (3'UTR, pos 3021) | No No |
| **HLA-B*51:01:01 (19)** | HLA-B*51:01:01:01 | HLA-B*51:01:01:03 | SNP T/C (3'UTR, pos 2978) | Yes (B*52:01:01:02) |
|  |  | HLA-B*51:01:01:04 | SNP C/T (3'UTR, pos 3435) SNP C/T (3'UTR, pos 3457) SNP A/T (3'UTR, pos 3507) SNP T/C (3'UTR, pos 3511) | Yes (B*51:01:01:05/06) Yes (B*51:01:01:05/06) Yes (B*44:03:01:10) Yes (B*51:01:01:09) |
|  |  | HLA-B*51:01:01:05 | SNP C/T (3'UTR, pos 3435) SNP C/T (3'UTR, pos 3457) SNP C/T (3'UTR, pos 3609) SNP G/C (3'UTR, pos 3698) SNP A/G (3'UTR, pos 3752) | Yes (B*51:01:01:04/06) Yes (B*51:01:01:04/06) Yes (B*51:01:01:06) Yes (B*51:01:01:06/09) No |
| **HLA-B*52:01:01 (8)** | HLA-B*52:01:01:01 | HLA-B*52:01:01:02 | SNP T/C (3'UTR, pos 2978) | Yes (B*51:01:01:03) |
|  |  | HLA-B*52:01:01:03 | SNP C/A (5'UTR, pos -180) | No |
| **HLA-B*56:01:01 (2)** | HLA-B*56:01:01:02 | HLA-B*56:01:01:03 | DEL G (3'UTR, pos 2708) | No |
|  |  |  | SNP C/A (3'UTR, pos 2988) | Yes (B*56:01:01:04) |
|  |  | HLA-B*56:01:01:04 | SNP C/A (3'UTR, pos 2988) | Yes (B*56:01:01:03) |
| **HLA-B*58:01:01 (3)** | HLA-B*58:01:01:01 | HLA-B*58:01:01:03 | SNP T/C (3'UTR, pos 3576) SNP G/C (3'UTR, pos 3698) | Yes (B*51:01:01:05) |
| **HLA-C*01:02:01 (11)** | HLA-C*01:02:01:01 | HLA-C*01:02:01:05 | SNP G/A (3'UTR, pos 3499, pos 3665) SNP G/T (3'UTR, pos 3590) SNP C/A (3'UTR, pos 3609) | No |
|  |  | HLA-C*01:02:01:06 | SNP T/C (3'UTR, pos 3005) | No |
| **HLA-C*02:02:02 (21)** | HLA-C*02:02:02:01 | HLA-C*02:02:02:03 | SNP T/C (3'UTR, pos 3005) INS C (3'UTR, pos 3037) | Yes (C*01:02:01:06; C*07:02:01:10) No |
|  |  | HLA-C*02:02:02:05 | SNP T/C (3'UTR, pos 3005) | Yes (C*01:02:01:06; C*07:02:01:10) |
| **HLA-C*03:03:01 (12)** | HLA-C*03:03:01:01 | HLA-C*03:03:01:09 | SNP T/C (3'UTR, pos 3440) | No |
| **HLA-C*03:04:01 (5)** | HLA-C*03:04:01:01 | HLA-C*03:04:01:02 | SNP G/C (3'UTR, pos 3033) | Yes (C*03:04:01:03) |
|  |  | HLA-C*04:01:01:14 | SNP A/C (3'UTR, pos 3095) SNP C/T (3'UTR, pos 3789) SNP C/T (3'UTR, pos 3801) SNP C/T (3'UTR, pos 3805) SNP A/G (3'UTR, pos 3806) | Yes (C*04:01:01:11) No No No No |
| **HLA-C*05:01:01 (11)** | HLA-C*05:01:01:02 | HLA-C*05:01:01:03 | SNP T/A (5'UTR, pos -136) | No |
| **HLA-C*07:01:01 (25)** | HLA-C*07:01:01:01 | HLA-C*07:01:01:08 | SNP G/A (3'UTR, pos 2987) | No |
|  |  | HLA-C*07:01:01:16 | SNP G/A (3'UTR, pos 3806) | Yes (C*04:01:01:01) |
| **HLA-C*07:02:01 (18)** | HLA-C*07:02:01:01 (4) | HLA-C*07:02:01:15 | SNP C/G (3'UTR, pos 3649) | No |
|  | HLA-C*07:02:01:03 (14) | HLA-C*07:02:01:09 | SNP G/A (3'UTR, pos 3058) | No |
|  |  | HLA-C*07:02:01:10 | SNP T/C (3'UTR, pos 3005) | Yes (C*01:02:01:06; C*02:02:02:03/05) |
|  |  | HLA-C*07:02:01:11 | SNP G/A (3'UTR, pos 2995) | No |
| **HLA-C*07:04:01 (11)** | HLA-C*07:04:01:01 | HLA-C*07:04:01:03 | SNP C/T (3'UTR, pos 3789) | Yes (C*04:01:01:14) |
|  |  |  | DEL GA (3'UTR, pos 3775) | No |
| **HLA-C*12:02:02 (8)** | HLA-C*12:02:02:01 | HLA-C*12:02:02:02 | SNP C/T (3'UTR, pos 3005) | Yes (C*01:02:01:01; C*02:02:02:01) |
| **HLA-C*12:03:01 (16)** | HLA-C*12:03:01:01 | HLA-C*12:03:01:06 | SNP T/C (3'UTR, pos 2988) | No |
| **HLA-C*14:02:01 (3)** | HLA-C*14:02:01:01 | HLA-C*14:02:01:04 | SNP T/C (3'UTR, pos 3789) | Yes (C*04:01:01:01) |
| **HLA-C*16:01:01 (1)** | HLA-C*16:01:01:01 | HLA-C*16:01:01:02 | SNP C/T (3'UTR, pos 2996) | No |
| **HLA-C*17:03:01 (2)** | HLA-C*17:03:01:01 | HLA-C*17:03:01:02 | SNP T/C (3'UTR, pos 3041) | No |
|  |  | HLA-C*17:03:01:03 | SNP A/G (3'UTR, pos 3020) | No |
| **HLA-DQB1*05:03:01 (13)** | HLA-DQB1*05:03:01:01 | HLA-DQB1*05:03:01:02 | SNP C/T (5'UTR, pos -156) | No |
| **HLA-DRB1*07:01:01 (22)** | HLA-DRB1*07:01:01:01 | HLA-DRB1*07:01:01:02 | SNP G/A (Intron 1, pos 4758)  SNP C/A (intron 1, pos 1181) | No No |
| **HLA-DRB1*15:01:01 (15)** | HLA-DRB1*15:01:01:03 | HLA-DRB1*15:01:01:04 | SNP C/T (Intron 4, pos 9210) | No |
|  |  |  | SNP T/C (Intron 1, pos 939) | No |
| **Observed first, second and third field ambiguities** | | | | |
|  | **Ambigous alleles** | |  |  |
| **HLA-DQB1*06:01:01/15 (7)** | HLA-DQB1*06:01:01 | DQB1*06:01:15 | SNP A/G (exon 1, pos 66) | No |
| **HLA-DRB1*12:01:01/12:10 (3)** | HLA-DRB1*12:01:01 | DRB1*12:10 | SNP A/G (exon 1, pos 40) | No |

**Supplementary Table 2. Summary of the Omixon Twin software sequencing metrics performed for each HLA locus after the MiSeq sequencing run. Data are given as median (2.5th-97.5th percentile).**

|  | **A** | **B** | **C** | **DQA1** | **DQB1** | **DRB1** | **DPB1** |
| --- | --- | --- | --- | --- | --- | --- | --- |
| **Coverage (%)** | 92.8 (90.4-97.3) | 74 (66.9-87.5) | 83.1 (70.9-92.3) | 95.5 (92.8-100) | 90.6 (90.3-91.7) | 45.3 (30-100) | 60.3 (60-60.5) |
| **Avg. depth** | 129 (81-253) | 112 (77-208) | 115 (77-222) | 153 (82-294) | 143 (83-264) | 66 (23-135) | 117 (44-169) |
| **Avg. coverage depth** | 144 (48-334) | 143 (42-314) | 155 (51-376) | 156 (70-308) | 156 (75-307) | 144 (14-395) | 187 (69-295) |
| **Detection (%)** | 100 | 100 | 100 | 100 | 100 | 100 | 100 |

**Supplementary Table 3. Summary of the Omixon Twin software quality control metrics across whole sample population (n=111)**

|  | **Reads kept.after.filtering** | | **Reads used.for.final.consensus.generation** | | **Average.fragment.size** | **Average.lenght.of.read** |
| --- | --- | --- | --- | --- | --- | --- |
|  | **Total read count** | **%** | **Total read count** | **%** | **Total read count** | **Total read count** |
| **mean** | 54359 | 40.2 | 49762 | 91.6 | 259 | 208 |
| **2.5-97.5th percentile** | 36245-65701 | 21-67.9 | 33530-60424 | 88.5-94.6 | 192-314 | 134-222 |

**Supplementary Table 4. Hardy-Weinberg equilibrium estimates**

| **Locus** | **#Genot** | **Obs.Het.** | **Exp.Het.** | **P-value** | **s.d.** | **Steps done** |
| --- | --- | --- | --- | --- | --- | --- |
| **A** | 111 | 0.83784 | 0.84261 | 0.4689 | 0.00021 | 1001000 |
| **B** | 111 | 0.97297 | 0.9572 | 0.81479 | 0.00018 | 1001000 |
| **C** | 111 | 0.98198 | 0.93094 | 0.66687 | 0.0002 | 1001000 |
| **DQA1** | 111 | 0.97297 | 0.94733 | 0.31264 | 0.00016 | 1001000 |
| **DQB1** | 111 | 0.94595 | 0.91643 | 0.20858 | 0.0002 | 1001000 |
| **DRB1** | 111 | 0.94595 | 0.94313 | 0.31573 | 0.00028 | 1001000 |

* Guo-Thompson exact test, p-value < 0.05

**Supplementary Table 5. Linkage disequilibrium significance level between each pair of HLA loci under study**

| **Locus pair** | **D'** | **Wn** | **p-value (1000 permutations)** |
| --- | --- | --- | --- |
| **A:C** | 0.60433 | 0.54081 | <0.001* |
| **A:B** | 0.68565 | 0.55001 | <0.001* |
| **A:DRB1** | 0.63659 | 0.50944 | 0.0701 |
| **A:DQA1** | 0.64472 | 0.51908 | 0.0100* |
| **A:DQB1** | 0.57485 | 0.39294 | 0.0631 |
| **C:B** | 0.92844 | 0.7992 | <0.001* |
| **C:DRB1** | 0.74948 | 0.55091 | <0.001* |
| **C:DQA1** | 0.75471 | 0.5248 | <0.001* |
| **C:DQB1** | 0.65469 | 0.46202 | <0.001* |
| **B:DRB1** | 0.81027 | 0.53657 | <0.001* |
| **B:DQA1** | 0.80021 | 0.50902 | <0.001* |
| **B:DQB1** | 0.7454 | 0.54651 | <0.001* |
| **DRB1:DQA1** | 0.97561 | 0.73319 | <0.001* |
| **DRB1:DQB1** | 0.9858 | 0.85548 | <0.001* |
| **DQA1:DQB1** | 0.99053 | 0.82538 | <0.001* |

**Supplementary Table 6. The complete list of HLA~A~B~C haplotypes in East Croatia blood donor volunteers (n=111).**

| **No.** | **HLA-A~B~C** | | | **Observed (n)** | **HF** | **cF** |
| --- | --- | --- | --- | --- | --- | --- |
| **1** | HLA-A*01:01:01:01 | HLA-B*08:01:01 | HLA-C*07:01:01 | 15.00 | 6.76% | 6.76% |
| **2** | HLA-A*11:01:01:01 | HLA-B*35:01:01 | HLA-C*04:01:01:01 | 8.00 | 3.60% | 10.36% |
| **3** | HLA-A*03:01:01:01 | HLA-B*07:02:01 | HLA-C*07:02:01 | 6.89 | 3.11% | 13.47% |
| **4** | HLA-A*02:01:01 | HLA-B*57:01:01:01 | HLA-C*06:02:01:01 | 6.00 | 2.70% | 16.17% |
| **5** | HLA-A*02:01:01 | HLA-B*27:02:01:01 | HLA-C*02:02:02 | 5.00 | 2.25% | 18.42% |
| **6** | HLA-A*02:01:01 | HLA-B*35:01:01 | HLA-C*04:01:01:06 | 5.00 | 2.25% | 20.67% |
| **7** | HLA-A*02:01:01 | HLA-B*35:03:01 | HLA-C*04:01:01:01 | 5.00 | 2.25% | 22.93% |
| **8** | HLA-A*02:01:01 | HLA-B*44:02:01:01 | HLA-C*05:01:01 | 5.00 | 2.25% | 25.18% |
| **9** | HLA-A*03:01:01:01 | HLA-B*35:03:01 | HLA-C*04:01:01:01 | 5.00 | 2.25% | 27.43% |
| **10** | HLA-A*02:01:01 | HLA-B*44:27:01 | HLA-C*07:04:01 | 4.00 | 1.80% | 29.23% |
| **11** | HLA-A*11:01:01:01 | HLA-B*52:01:01 | HLA-C*12:02:02 | 4.00 | 1.80% | 31.03% |
| **12** | HLA-A*02:01:01 | HLA-B*27:05:02 | HLA-C*02:02:02 | 3.89 | 1.75% | 32.79% |
| **13** | HLA-A*02:01:01 | HLA-B*18:01:01 | HLA-C*07:01:01 | 3.00 | 1.35% | 34.14% |
| **14** | HLA-A*02:01:01 | HLA-B*52:01:01 | HLA-C*12:02:02 | 3.00 | 1.35% | 35.49% |
| **15** | HLA-A*03:01:01:01 | HLA-B*15:01:01:01 | HLA-C*07:04:01 | 3.00 | 1.35% | 36.84% |
| **16** | HLA-A*23:01:01:01 | HLA-B*44:03:01 | HLA-C*04:01:01:01 | 3.00 | 1.35% | 38.19% |
| **17** | HLA-A*24:02:01:01 | HLA-B*13:02:01:01 | HLA-C*06:02:01:01 | 3.00 | 1.35% | 39.54% |
| **18** | HLA-A*24:02:01:01 | HLA-B*35:02:01 | HLA-C*04:01:01:06 | 3.00 | 1.35% | 40.90% |
| **19** | HLA-A*24:02:01:01 | HLA-B*51:01:01 | HLA-C*15:02:01:01 | 3.00 | 1.35% | 42.25% |
| **20** | HLA-A*02:01:01 | HLA-B*07:02:01 | HLA-C*07:02:01 | 2.88 | 1.30% | 43.54% |
| **21** | HLA-A*01:01:01:01 | HLA-B*27:02:01:01 | HLA-C*02:02:02 | 2.00 | 0.90% | 44.44% |
| **22** | HLA-A*01:01:01:01 | HLA-B*55:01:01 | HLA-C*01:02:01 | 2.00 | 0.90% | 45.35% |
| **23** | HLA-A*01:01:01:01 | HLA-B*55:01:01 | HLA-C*03:03:01 | 2.00 | 0.90% | 46.25% |
| **24** | HLA-A*02:01:01 | HLA-B*15:17:01:01 | HLA-C*07:01:02 | 2.00 | 0.90% | 47.15% |
| **25** | HLA-A*02:01:01 | HLA-B*18:01:01 | HLA-C*12:03:01 | 2.00 | 0.90% | 48.05% |
| **26** | HLA-A*02:01:01 | HLA-B*27:05:02 | HLA-C*01:02:01 | 2.00 | 0.90% | 48.95% |
| **27** | HLA-A*02:01:01 | HLA-B*38:01:01 | HLA-C*12:03:01 | 2.00 | 0.90% | 49.85% |
| **28** | HLA-A*02:01:01 | HLA-B*51:01:01 | HLA-C*02:02:02 | 2.00 | 0.90% | 50.75% |
| **29** | HLA-A*02:01:01 | HLA-B*51:01:01 | HLA-C*14:02:01 | 2.00 | 0.90% | 51.65% |
| **30** | HLA-A*02:01:01 | HLA-B*55:01:01 | HLA-C*03:03:01 | 2.00 | 0.90% | 52.55% |
| **31** | HLA-A*03:01:01:01 | HLA-B*44:27:01 | HLA-C*07:04:01 | 2.00 | 0.90% | 53.45% |
| **32** | HLA-A*03:01:01:01 | HLA-B*51:01:01 | HLA-C*15:02:01:01 | 2.00 | 0.90% | 54.35% |
| **33** | HLA-A*11:01:01:01 | HLA-B*35:03:01 | HLA-C*04:01:01:01 | 2.00 | 0.90% | 55.26% |
| **34** | HLA-A*24:02:01:01 | HLA-B*40:01:02 | HLA-C*03:04:01 | 2.00 | 0.90% | 56.16% |
| **35** | HLA-A*25:01:01:01 | HLA-B*18:01:01 | HLA-C*12:03:01 | 2.00 | 0.90% | 57.06% |
| **36** | HLA-A*32:01:01:01 | HLA-B*40:02:01 | HLA-C*02:02:02 | 2.00 | 0.90% | 57.96% |
| **37** | HLA-A*32:01:01:01 | HLA-B*44:02:01:01 | HLA-C*05:01:01 | 2.00 | 0.90% | 58.86% |
| **38** | HLA-A*66:01:01:01 | HLA-B*41:02:01 | HLA-C*17:03:01 | 2.00 | 0.90% | 59.76% |
| **39** | HLA-A*24:02:01:01 | HLA-B*51:01:01 | HLA-C*01:02:01 | 1.77 | 0.80% | 60.56% |
| **40** | HLA-A*02:01:01 | HLA-B*51:01:01 | HLA-C*01:02:01 | 1.23 | 0.55% | 61.11% |
| **41** | HLA-A*24:02:01:01 | HLA-B*07:02:01 | HLA-C*07:02:01 | 1.23 | 0.55% | 61.66% |
| **42** | HLA-A*03:01:01:01 | HLA-B*27:05:02 | HLA-C*02:02:02 | 1.11 | 0.50% | 62.16% |
| **43** | HLA-A*01:01:01:01 | HLA-B*13:02:01:01 | HLA-C*06:02:01:01 | 1.00 | 0.45% | 62.61% |
| **44** | HLA-A*01:01:01:01 | HLA-B*18:01:01 | HLA-C*12:03:01 | 1.00 | 0.45% | 63.06% |
| **45** | HLA-A*01:01:01:01 | HLA-B*39:01:01 | HLA-C*12:03:01 | 1.00 | 0.45% | 63.51% |
| **46** | HLA-A*01:01:01:01 | HLA-B*40:02:01 | HLA-C*02:02:02 | 1.00 | 0.45% | 63.96% |
| **47** | HLA-A*01:01:01:01 | HLA-B*54:01:01 | HLA-C*06:02:01:01 | 1.00 | 0.45% | 64.42% |
| **48** | HLA-A*01:01:01:01 | HLA-B*58:01:01 | HLA-C*04:01:01:06 | 1.00 | 0.45% | 64.87% |
| **49** | HLA-A*02:01:01 | HLA-B*07:06:01 | HLA-C*15:05:02 | 1.00 | 0.45% | 65.32% |
| **50** | HLA-A*02:01:01 | HLA-B*08:01:01 | HLA-C*07:01:01 | 1.00 | 0.45% | 65.77% |
| **51** | HLA-A*02:01:01 | HLA-B*13:02:01:01 | HLA-C*07:02:01 | 1.00 | 0.45% | 66.22% |
| **52** | HLA-A*02:01:01 | HLA-B*15:01:01:01 | HLA-C*03:03:01 | 1.00 | 0.45% | 66.67% |
| **53** | HLA-A*02:01:01 | HLA-B*15:01:06 | HLA-C*03:03:01 | 1.00 | 0.45% | 67.12% |
| **54** | HLA-A*02:01:01 | HLA-B*18:01:01 | HLA-C*07:01:09 | 1.00 | 0.45% | 67.57% |
| **55** | HLA-A*02:01:01 | HLA-B*18:01:01 | HLA-C*07:02:01 | 1.00 | 0.45% | 68.02% |
| **56** | HLA-A*02:01:01 | HLA-B*35:03:01 | HLA-C*12:03:01 | 1.00 | 0.45% | 68.47% |
| **57** | HLA-A*02:01:01 | HLA-B*37:01:01:01 | HLA-C*06:02:01:01 | 1.00 | 0.45% | 68.92% |
| **58** | HLA-A*02:01:01 | HLA-B*39:01:01 | HLA-C*06:02:01:01 | 1.00 | 0.45% | 69.37% |
| **59** | HLA-A*02:01:01 | HLA-B*40:01:02 | HLA-C*03:04:01 | 1.00 | 0.45% | 69.82% |
| **60** | HLA-A*02:01:01 | HLA-B*44:05:01 | HLA-C*02:02:02 | 1.00 | 0.45% | 70.27% |
| **61** | HLA-A*02:01:01 | HLA-B*51:01:01 | HLA-C*07:02:01 | 1.00 | 0.45% | 70.72% |
| **62** | HLA-A*02:01:01 | HLA-B*51:01:01 | HLA-C*16:02:01 | 1.00 | 0.45% | 71.17% |
| **63** | HLA-A*03:01:01:01 | HLA-B*15:01:01:01 | HLA-C*03:03:01 | 1.00 | 0.45% | 71.62% |
| **64** | HLA-A*03:01:01:01 | HLA-B*15:17:01:01 | HLA-C*07:01:02 | 1.00 | 0.45% | 72.07% |
| **65** | HLA-A*03:01:01:01 | HLA-B*27:02:01:04 | HLA-C*05:01:01 | 1.00 | 0.45% | 72.52% |
| **66** | HLA-A*03:01:01:01 | HLA-B*35:01:01 | HLA-C*04:01:01:01 | 1.00 | 0.45% | 72.97% |
| **67** | HLA-A*03:01:01:01 | HLA-B*44:02:01:01 | HLA-C*05:01:01 | 1.00 | 0.45% | 73.43% |
| **68** | HLA-A*03:01:01:01 | HLA-B*56:01:01 | HLA-C*16:01:01 | 1.00 | 0.45% | 73.88% |
| **69** | HLA-A*03:01:01:03 | HLA-B*51:01:01 | HLA-C*01:02:01 | 1.00 | 0.45% | 74.33% |
| **70** | HLA-A*03:01:01:05 | HLA-B*51:01:01 | HLA-C*03:03:01 | 1.00 | 0.45% | 74.78% |
| **71** | HLA-A*11:01:01:01 | HLA-B*07:02:01 | HLA-C*07:02:01 | 1.00 | 0.45% | 75.23% |
| **72** | HLA-A*11:01:01:01 | HLA-B*07:05:01 | HLA-C*15:05:02 | 1.00 | 0.45% | 75.68% |
| **73** | HLA-A*11:01:01:01 | HLA-B*08:01:01 | HLA-C*07:01:01 | 1.00 | 0.45% | 76.13% |
| **74** | HLA-A*11:01:01:01 | HLA-B*15:01:01:01 | HLA-C*03:03:01 | 1.00 | 0.45% | 76.58% |
| **75** | HLA-A*11:01:01:01 | HLA-B*18:03 | HLA-C*02:02:02 | 1.00 | 0.45% | 77.03% |
| **76** | HLA-A*11:01:01:01 | HLA-B*35:08:01:01 | HLA-C*05:01:01 | 1.00 | 0.45% | 77.48% |
| **77** | HLA-A*11:01:01:01 | HLA-B*51:01:01 | HLA-C*04:01:01:01 | 1.00 | 0.45% | 77.93% |
| **78** | HLA-A*11:01:01:01 | HLA-B*57:01:01:01 | HLA-C*06:02:01:01 | 1.00 | 0.45% | 78.38% |
| **79** | HLA-A*23:01:01:01 | HLA-B*13:02:01:01 | HLA-C*06:02:01:01 | 1.00 | 0.45% | 78.83% |
| **80** | HLA-A*23:01:01:01 | HLA-B*18:03 | HLA-C*04:01:01:01 | 1.00 | 0.45% | 79.28% |
| **81** | HLA-A*24:02:01:01 | HLA-B*07:02:01 | HLA-C*12:03:01 | 1.00 | 0.45% | 79.73% |
| **82** | HLA-A*24:02:01:01 | HLA-B*08:01:01 | HLA-C*07:02:01 | 1.00 | 0.45% | 80.18% |
| **83** | HLA-A*24:02:01:01 | HLA-B*14:02:01:01 | HLA-C*08:02:01:01 | 1.00 | 0.45% | 80.63% |
| **84** | HLA-A*24:02:01:01 | HLA-B*15:10:01 | HLA-C*03:03:01 | 1.00 | 0.45% | 81.08% |
| **85** | HLA-A*24:02:01:01 | HLA-B*18:01:01 | HLA-C*07:01:01 | 1.00 | 0.45% | 81.53% |
| **86** | HLA-A*24:02:01:01 | HLA-B*35:01:01 | HLA-C*04:01:01:01 | 1.00 | 0.45% | 81.98% |
| **87** | HLA-A*24:02:01:01 | HLA-B*35:02:01 | HLA-C*02:02:02 | 1.00 | 0.45% | 82.44% |
| **88** | HLA-A*24:02:01:01 | HLA-B*39:01:01 | HLA-C*07:02:01 | 1.00 | 0.45% | 82.89% |
| **89** | HLA-A*24:02:01:01 | HLA-B*41:02:01 | HLA-C*12:03:01 | 1.00 | 0.45% | 83.34% |
| **90** | HLA-A*24:02:01:01 | HLA-B*44:02:01:01 | HLA-C*04:01:01:01 | 1.00 | 0.45% | 83.79% |
| **91** | HLA-A*24:02:01:01 | HLA-B*49:01:01 | HLA-C*07:01:01 | 1.00 | 0.45% | 84.24% |
| **92** | HLA-A*24:02:01:01 | HLA-B*52:01:01 | HLA-C*12:02:02 | 1.00 | 0.45% | 84.69% |
| **93** | HLA-A*24:02:01:05 | HLA-B*15:01:01:01 | HLA-C*03:03:01 | 1.00 | 0.45% | 85.14% |
| **94** | HLA-A*24:02:01:05 | HLA-B*27:05:02 | HLA-C*07:01:01 | 1.00 | 0.45% | 85.59% |
| **95** | HLA-A*25:01:01:01 | HLA-B*15:01:01:01 | HLA-C*03:03:01 | 1.00 | 0.45% | 86.04% |
| **96** | HLA-A*25:01:01:01 | HLA-B*44:02:01:03 | HLA-C*07:04:01 | 1.00 | 0.45% | 86.49% |
| **97** | HLA-A*25:01:01:01 | HLA-B*55:01:01 | HLA-C*03:04:01 | 1.00 | 0.45% | 86.94% |
| **98** | HLA-A*26:01:01:01 | HLA-B*27:02:01:01 | HLA-C*02:02:02 | 1.00 | 0.45% | 87.39% |
| **99** | HLA-A*26:01:01:01 | HLA-B*38:01:01 | HLA-C*12:03:01 | 1.00 | 0.45% | 87.84% |
| **100** | HLA-A*26:01:01:01 | HLA-B*55:01:01 | HLA-C*15:02:01:01 | 1.00 | 0.45% | 88.29% |
| **101** | HLA-A*26:01:01:01 | HLA-B*56:01:01 | HLA-C*01:02:01 | 1.00 | 0.45% | 88.74% |
| **102** | HLA-A*26:01:01:06 | HLA-B*51:01:01 | HLA-C*14:02:01 | 1.00 | 0.45% | 89.19% |
| **103** | HLA-A*26:08 | HLA-B*18:01:01 | HLA-C*07:01:01 | 1.00 | 0.45% | 89.64% |
| **104** | HLA-A*29:01:01:01 | HLA-B*50:01:01:01 | HLA-C*06:02:01:02 | 1.00 | 0.45% | 90.09% |
| **105** | HLA-A*29:02:01:01 | HLA-B*44:03:01 | HLA-C*01:02:01 | 1.00 | 0.45% | 90.54% |
| **106** | HLA-A*29:02:01:01 | HLA-B*58:01:01 | HLA-C*07:18 | 1.00 | 0.45% | 90.99% |
| **107** | HLA-A*30:01:01 | HLA-B*15:01:01:01 | HLA-C*03:04:02 | 1.00 | 0.45% | 91.45% |
| **108** | HLA-A*30:01:01 | HLA-B*35:01:01 | HLA-C*04:01:01:01 | 1.00 | 0.45% | 91.90% |
| **109** | HLA-A*30:01:01 | HLA-B*35:03:01 | HLA-C*12:03:01 | 1.00 | 0.45% | 92.35% |
| **110** | HLA-A*31:01:02:01 | HLA-B*07:02:01 | HLA-C*07:02:01 | 1.00 | 0.45% | 92.80% |
| **111** | HLA-A*31:01:02:01 | HLA-B*15:17:01:01 | HLA-C*07:01:02 | 1.00 | 0.45% | 93.25% |
| **112** | HLA-A*31:01:02:01 | HLA-B*35:08:01:01 | HLA-C*07:18 | 1.00 | 0.45% | 93.70% |
| **113** | HLA-A*31:01:02:01 | HLA-B*37:01:01:01 | HLA-C*01:02:01 | 1.00 | 0.45% | 94.15% |
| **114** | HLA-A*31:01:02:01 | HLA-B*49:01:01 | HLA-C*07:01:01 | 1.00 | 0.45% | 94.60% |
| **115** | HLA-A*31:01:02:04 | HLA-B*07:02:01 | HLA-C*12:03:01 | 1.00 | 0.45% | 95.05% |
| **116** | HLA-A*32:01:01:01 | HLA-B*27:05:02 | HLA-C*03:04:01 | 1.00 | 0.45% | 95.50% |
| **117** | HLA-A*32:01:01:01 | HLA-B*35:01:01 | HLA-C*04:01:01:01 | 1.00 | 0.45% | 95.95% |
| **118** | HLA-A*32:01:01:01 | HLA-B*38:01:01 | HLA-C*12:03:01 | 1.00 | 0.45% | 96.40% |
| **119** | HLA-A*32:01:01:01 | HLA-B*39:01:01 | HLA-C*12:03:01 | 1.00 | 0.45% | 96.85% |
| **120** | HLA-A*33:01:01:01 | HLA-B*14:02:01:01 | HLA-C*08:02:01:01 | 1.00 | 0.45% | 97.30% |
| **121** | HLA-A*33:03:01:02 | HLA-B*58:01:01 | HLA-C*03:02:02:01 | 1.00 | 0.45% | 97.75% |
| **122** | HLA-A*68:01:01:02 | HLA-B*35:03:01 | HLA-C*05:01:01 | 1.00 | 0.45% | 98.20% |
| **123** | HLA-A*68:01:02:02 | HLA-B*35:01:01 | HLA-C*07:04:01 | 1.00 | 0.45% | 98.65% |
| **124** | HLA-A*68:01:02:02 | HLA-B*44:03:01 | HLA-C*04:01:01:01 | 1.00 | 0.45% | 99.10% |
| **125** | HLA-A*68:01:02:02 | HLA-B*51:01:01 | HLA-C*15:02:01:01 | 1.00 | 0.45% | 99.55% |
| **126** | HLA-A*68:02:01:01 | HLA-B*14:02:01:01 | HLA-C*08:02:01:01 | 1.00 | 0.45% | 100.00% |

**Supplementary Table 7. The complete list of HLA-DQA1~DQB1~DRB1 haplotypes in East Croatia blood donor volunteers (n=111).**

| **No.** | **HLA-DQA1~DQB1~DRB1** | | | **Observed (n)** | | **HF** | **cF** |
| --- | --- | --- | --- | --- | --- | --- | --- |
| **1** | HLA-DQA1*01:02:02 | HLA-DQB1*05:02:01:01 | HLA-DRB1*16:01:01 | | 30.00 | 13.51% | 13.51% |
| **2** | HLA-DQA1*02:01:01:01 | HLA-DQB1*02:02:01:01 | HLA-DRB1*07:01:01 | | 11.82 | 5.32% | 18.84% |
| **3** | HLA-DQA1*05:01:01:02 | HLA-DQB1*02:01:01 | HLA-DRB1*03:01:01:01 | | 11.00 | 4.96% | 23.79% |
| **4** | HLA-DQA1*01:02:01:01 | HLA-DQB1*06:02:01:01 | HLA-DRB1*15:01:01 | | 10.00 | 4.50% | 28.30% |
| **5** | HLA-DQA1*01:01:01:01 | HLA-DQB1*05:01:01:03 | HLA-DRB1*01:01:01 | | 9.00 | 4.05% | 32.35% |
| **6** | HLA-DQA1*01:01:01:03 | HLA-DQB1*05:01:01:03 | HLA-DRB1*01:01:01 | | 7.00 | 3.15% | 35.50% |
| **7** | HLA-DQA1*01:03:01:01 | HLA-DQB1*06:01:01/15 | HLA-DRB1*15:02:01:01 | | 7.00 | 3.15% | 38.66% |
| **8** | HLA-DQA1*01:03:01:02 | HLA-DQB1*06:03:01:01 | HLA-DRB1*13:01:01:01 | | 7.00 | 3.15% | 41.81% |
| **9** | HLA-DQA1*01:04:01:01 | HLA-DQB1*05:03:01 | HLA-DRB1*14:54:01:02 | | 6.00 | 2.70% | 44.51% |
| **10** | HLA-DQA1*05:05:01:09 | HLA-DQB1*03:01:01:03 | HLA-DRB1*11:04:01 | | 5.00 | 2.25% | 46.76% |
| **11** | HLA-DQA1*01:01:01:02 | HLA-DQB1*05:01:01:03 | HLA-DRB1*01:01:01 | | 4.00 | 1.80% | 48.57% |
| **12** | HLA-DQA1*01:02:01:04 | HLA-DQB1*06:04:01 | HLA-DRB1*13:02:01:02 | | 4.00 | 1.80% | 50.37% |
| **13** | HLA-DQA1*01:03:01:02 | HLA-DQB1*06:03:01:01 | HLA-DRB1*13:01:01:02 | | 4.00 | 1.80% | 52.17% |
| **14** | HLA-DQA1*04:01:01:03 | HLA-DQB1*04:02:01:01 | HLA-DRB1*08:01:01 | | 4.00 | 1.80% | 53.97% |
| **15** | HLA-DQA1*05:01:01:03 | HLA-DQB1*02:01:01 | HLA-DRB1*03:01:01:03 | | 4.00 | 1.80% | 55.77% |
| **16** | HLA-DQA1*02:01:01:02 | HLA-DQB1*03:03:02:01 | HLA-DRB1*07:01:01 | | 3.82 | 1.72% | 57.49% |
| **17** | HLA-DQA1*02:01:01:01 | HLA-DQB1*03:03:02:01 | HLA-DRB1*07:01:01 | | 3.18 | 1.43% | 58.93% |
| **18** | HLA-DQA1*02:01:01:02 | HLA-DQB1*02:02:01:01 | HLA-DRB1*07:01:01 | | 3.18 | 1.43% | 60.36% |
| **19** | HLA-DQA1*01:01:01:05 | HLA-DQB1*05:01:01:03 | HLA-DRB1*01:01:01 | | 3.00 | 1.35% | 61.71% |
| **20** | HLA-DQA1*01:02:01:05 | HLA-DQB1*06:02:01:01 | HLA-DRB1*15:01:01 | | 3.00 | 1.35% | 63.06% |
| **21** | HLA-DQA1*03:01:01 | HLA-DQB1*03:02:01:01 | HLA-DRB1*04:01:01:03 | | 3.00 | 1.35% | 64.41% |
| **22** | HLA-DQA1*03:01:01 | HLA-DQB1*03:02:01:01 | HLA-DRB1*04:02:01 | | 3.00 | 1.35% | 65.77% |
| **23** | HLA-DQA1*03:01:01 | HLA-DQB1*03:02:01:01 | HLA-DRB1*04:03:01:01 | | 3.00 | 1.35% | 67.12% |
| **24** | HLA-DQA1*05:05:01:02 | HLA-DQB1*03:01:01:03 | HLA-DRB1*11:01:01:01 | | 3.00 | 1.35% | 68.47% |
| **25** | HLA-DQA1*05:05:01:04 | HLA-DQB1*03:01:01:03 | HLA-DRB1*11:01:01:01 | | 3.00 | 1.35% | 69.82% |
| **26** | HLA-DQA1*05:05:01:05 | HLA-DQB1*03:01:01:03 | HLA-DRB1*13:03:01 | | 3.00 | 1.35% | 71.17% |
| **27** | HLA-DQA1*05:05:01:09 | HLA-DQB1*03:01:01:03 | HLA-DRB1*11:01:01:03 | | 3.00 | 1.35% | 72.52% |
| **28** | HLA-DQA1*01:02:02 | HLA-DQB1*05:02:01:01 | HLA-DRB1*15:01:01 | | 2.00 | 0.90% | 73.42% |
| **29** | HLA-DQA1*01:02:02 | HLA-DQB1*05:02:01:01 | HLA-DRB1*16:02:01:02 | | 2.00 | 0.90% | 74.33% |
| **30** | HLA-DQA1*01:03:01:02 | HLA-DQB1*06:03:01:02 | HLA-DRB1*13:01:01:02 | | 2.00 | 0.90% | 75.23% |
| **31** | HLA-DQA1*01:04:01:02 | HLA-DQB1*05:03:01 | HLA-DRB1*14:54:01:02 | | 2.00 | 0.90% | 76.13% |
| **32** | HLA-DQA1*01:05:01 | HLA-DQB1*05:01:01:05 | HLA-DRB1*10:01:01:03 | | 2.00 | 0.90% | 77.03% |
| **33** | HLA-DQA1*03:01:01 | HLA-DQB1*03:02:01:01 | HLA-DRB1*04:01:01:01 | | 2.00 | 0.90% | 77.93% |
| **34** | HLA-DQA1*03:01:01 | HLA-DQB1*03:02:01:01 | HLA-DRB1*08:01:01 | | 2.00 | 0.90% | 78.83% |
| **35** | HLA-DQA1*03:02:01:01 | HLA-DQB1*03:03:02:02 | HLA-DRB1*09:01:02 | | 2.00 | 0.90% | 79.73% |
| **36** | HLA-DQA1*04:02 | HLA-DQB1*04:02:01:01 | HLA-DRB1*08:01:01 | | 2.00 | 0.90% | 80.63% |
| **37** | HLA-DQA1*05:05:01:01 | HLA-DQB1*03:01:01:03 | HLA-DRB1*11:04:01 | | 2.00 | 0.90% | 81.53% |
| **38** | HLA-DQA1*05:05:01:04 | HLA-DQB1*03:01:01:03 | HLA-DRB1*11:03:01 | | 2.00 | 0.90% | 82.43% |
| **39** | HLA-DQA1*05:05:01:04 | HLA-DQB1*03:01:01:03 | HLA-DRB1*11:04:01 | | 2.00 | 0.90% | 83.33% |
| **40** | HLA-DQA1*05:05:01:09 | HLA-DQB1*03:01:01:03 | HLA-DRB1*11:01:01:01 | | 2.00 | 0.90% | 84.23% |
| **41** | HLA-DQA1*01:01:02 | HLA-DQB1*05:01:01:01 | HLA-DRB1*01:02:01 | | 1.00 | 0.45% | 84.69% |
| **42** | HLA-DQA1*01:02:01:03 | HLA-DQB1*05:04 | HLA-DRB1*01:01:01 | | 1.00 | 0.45% | 85.14% |
| **43** | HLA-DQA1*01:02:01:04 | HLA-DQB1*06:03:01:02 | HLA-DRB1*13:02:01:02 | | 1.00 | 0.45% | 85.59% |
| **44** | HLA-DQA1*01:02:02 | HLA-DQB1*05:02:01:01 | HLA-DRB1*16:01:02 | | 1.00 | 0.45% | 86.04% |
| **45** | HLA-DQA1*01:03:01:06 | HLA-DQB1*06:03:01:01 | HLA-DRB1*13:01:01:02 | | 1.00 | 0.45% | 86.49% |
| **46** | HLA-DQA1*01:04:01:02 | HLA-DQB1*05:03:01 | HLA-DRB1*14:01:01 | | 1.00 | 0.45% | 86.94% |
| **47** | HLA-DQA1*01:04:01:02 | HLA-DQB1*05:03:01 | HLA-DRB1*14:05:01:02 | | 1.00 | 0.45% | 87.39% |
| **48** | HLA-DQA1*01:04:01:02 | HLA-DQB1*05:03:01 | HLA-DRB1*14:54:01:01 | | 1.00 | 0.45% | 87.84% |
| **49** | HLA-DQA1*01:04:01:03 | HLA-DQB1*05:03:01 | HLA-DRB1*14:54:01:02 | | 1.00 | 0.45% | 88.29% |
| **50** | HLA-DQA1*01:04:01:04 | HLA-DQB1*05:03:01 | HLA-DRB1*14:01:01 | | 1.00 | 0.45% | 88.74% |
| **51** | HLA-DQA1*01:05:01 | HLA-DQB1*05:01:01:02 | HLA-DRB1*10:01:01:03 | | 1.00 | 0.45% | 89.19% |
| **52** | HLA-DQA1*01:05:01 | HLA-DQB1*05:01:01:05 | HLA-DRB1*10:01:01:01 | | 1.00 | 0.45% | 89.64% |
| **53** | HLA-DQA1*01:10 | HLA-DQB1*06:03:01:01 | HLA-DRB1*13:01:01:02 | | 1.00 | 0.45% | 90.09% |
| **54** | HLA-DQA1*03:01:01 | HLA-DQB1*03:02:01:01 | HLA-DRB1*04:01:01:02 | | 1.00 | 0.45% | 90.54% |
| **55** | HLA-DQA1*03:01:01 | HLA-DQB1*03:02:01:01 | HLA-DRB1*04:15 | | 1.00 | 0.45% | 90.99% |
| **56** | HLA-DQA1*03:01:01 | HLA-DQB1*03:02:01:02 | HLA-DRB1*04:04:01 | | 1.00 | 0.45% | 91.44% |
| **57** | HLA-DQA1*03:03:01:01 | HLA-DQB1*03:01:01:01 | HLA-DRB1*04:08:01 | | 1.00 | 0.45% | 91.89% |
| **58** | HLA-DQA1*03:03:01:01 | HLA-DQB1*03:01:01:10 | HLA-DRB1*04:08:01 | | 1.00 | 0.45% | 92.34% |
| **59** | HLA-DQA1*03:03:01:01 | HLA-DQB1*03:02:01:01 | HLA-DRB1*04:05:01:03 | | 1.00 | 0.45% | 92.79% |
| **60** | HLA-DQA1*04:01:02:01 | HLA-DQB1*04:02:01:01 | HLA-DRB1*08:04:01 | | 1.00 | 0.45% | 93.24% |
| **61** | HLA-DQA1*05:01:01:02 | HLA-DQB1*02:01:01 | HLA-DRB1*03:01:01:02 | | 1.00 | 0.45% | 93.70% |
| **62** | HLA-DQA1*05:01:01:03 | HLA-DQB1*02:01:01 | HLA-DRB1*03:01:01:01 | | 1.00 | 0.45% | 94.15% |
| **63** | HLA-DQA1*05:05:01:01 | HLA-DQB1*03:01:01:02 | HLA-DRB1*11:04:01 | | 1.00 | 0.45% | 94.60% |
| **64** | HLA-DQA1*05:05:01:01 | HLA-DQB1*03:01:01:03 | HLA-DRB1*11:01:01:01 | | 1.00 | 0.45% | 95.05% |
| **65** | HLA-DQA1*05:05:01:01 | HLA-DQB1*03:01:01:03 | HLA-DRB1*11:01:01:03 | | 1.00 | 0.45% | 95.50% |
| **66** | HLA-DQA1*05:05:01:01 | HLA-DQB1*03:01:01:03 | HLA-DRB1*11:03:01 | | 1.00 | 0.45% | 95.95% |
| **67** | HLA-DQA1*05:05:01:02 | HLA-DQB1*03:01:01:03 | HLA-DRB1*13:05:01 | | 1.00 | 0.45% | 96.40% |
| **68** | HLA-DQA1*05:05:01:03 | HLA-DQB1*03:01:01:05 | HLA-DRB1*12:01:01/12:10 | | 1.00 | 0.45% | 96.85% |
| **69** | HLA-DQA1*05:05:01:04 | HLA-DQB1*03:01:01:03 | HLA-DRB1*11:01:01:03 | | 1.00 | 0.45% | 97.30% |
| **70** | HLA-DQA1*05:05:01:04 | HLA-DQB1*03:01:01:19 | HLA-DRB1*11:01:08 | | 1.00 | 0.45% | 97.75% |
| **71** | HLA-DQA1*05:05:01:05 | HLA-DQB1*03:01:01:05 | HLA-DRB1*12:01:01/12:10 | | 1.00 | 0.45% | 98.20% |
| **72** | HLA-DQA1*05:05:01:06 | HLA-DQB1*03:01:01:03 | HLA-DRB1*04:05:01:03 | | 1.00 | 0.45% | 98.65% |
| **73** | HLA-DQA1*05:05:01:06 | HLA-DQB1*03:01:01:05 | HLA-DRB1*12:01:01/12:10 | | 1.00 | 0.45% | 99.10% |
| **74** | HLA-DQA1*05:05:01:08 | HLA-DQB1*03:01:01:03 | HLA-DRB1*11:01:01:01 | | 1.00 | 0.45% | 99.55% |
| **75** | HLA-DQA1*05:05:01:08 | HLA-DQB1*03:01:01:03 | HLA-DRB1*11:01:01:03 | | 1.00 | 0.45% | 100.00% |

**Supplementary Table 8. The complete list of HLA-A~B~C~DRB1~DQA1~DQB1 extended haplotypes in East Croatian blood donor volunteers (n=111)**

| **No.** | **HLA-A~B~C~DQA1~DQB1~DRB1** | | | | | | **n** | **HF** | **cF** |
| --- | --- | --- | --- | --- | --- | --- | --- | --- | --- |
| **1** | HLA-A*01:01:01:01 | HLA-B*08:01:01 | HLA-C*07:01:01 | HLA-DQA1*05:01:01:02 | HLA-DQB1*02:01:01 | HLA-DRB1*03:01:01:01 | 8.00 | 3.60% | 3.60% |
| **2** | HLA-A*02:01:01 | HLA-B*18:01:01 | HLA-C*07:01:01 | HLA-DQA1*05:05:01:09 | HLA-DQB1*03:01:01:03 | HLA-DRB1*11:04:01 | 4.00 | 1.80% | 5.41% |
| **3** | HLA-A*02:01:01 | HLA-B*27:02:01:01 | HLA-C*02:02:02 | HLA-DQA1*01:02:02 | HLA-DQB1*05:02:01:01 | HLA-DRB1*16:01:01 | 4.00 | 1.80% | 7.21% |
| **4** | HLA-A*03:01:01:01 | HLA-B*07:02:01 | HLA-C*07:02:01 | HLA-DQA1*01:02:02 | HLA-DQB1*05:02:01:01 | HLA-DRB1*16:01:01 | 4.00 | 1.80% | 9.01% |
| **5** | HLA-A*11:01:01:01 | HLA-B*35:01:01 | HLA-C*04:01:01:01 | HLA-DQA1*01:02:02 | HLA-DQB1*05:02:01:01 | HLA-DRB1*16:01:01 | 4.00 | 1.80% | 10.81% |
| **6** | HLA-A*02:01:01 | HLA-B*13:02:01:01 | HLA-C*06:02:01:01 | HLA-DQA1*02:01:01:01 | HLA-DQB1*02:02:01:01 | HLA-DRB1*07:01:01 | 3.00 | 1.35% | 12.16% |
| **7** | HLA-A*02:01:01 | HLA-B*44:27:01 | HLA-C*07:04:01 | HLA-DQA1*01:02:02 | HLA-DQB1*05:02:01:01 | HLA-DRB1*16:01:01 | 3.00 | 1.35% | 13.51% |
| **8** | HLA-A*02:01:01 | HLA-B*52:01:01 | HLA-C*12:02:02 | HLA-DQA1*01:03:01:01 | HLA-DQB1*06:01:01/15 | HLA-DRB1*15:02:01:01 | 3.00 | 1.35% | 14.87% |
| **9** | HLA-A*11:01:01:01 | HLA-B*35:01:01 | HLA-C*04:01:01:01 | HLA-DQA1*01:01:01:01 | HLA-DQB1*05:01:01:03 | HLA-DRB1*01:01:01 | 3.00 | 1.35% | 16.22% |
| **10** | HLA-A*24:02:01:01 | HLA-B*13:02:01:01 | HLA-C*06:02:01:01 | HLA-DQA1*02:01:01:01 | HLA-DQB1*02:02:01:01 | HLA-DRB1*07:01:01 | 3.00 | 1.35% | 17.57% |
| **11** | HLA-A*02:01:01 | HLA-B*35:03:01 | HLA-C*04:01:01:01 | HLA-DQA1*01:02:02 | HLA-DQB1*05:02:01:01 | HLA-DRB1*16:01:01 | 2.00 | 0.90% | 18.47% |
| **12** | HLA-A*02:01:01 | HLA-B*57:01:01:01 | HLA-C*06:02:01:01 | HLA-DQA1*02:01:01:01 | HLA-DQB1*03:03:02:01 | HLA-DRB1*07:01:01 | 2.00 | 0.90% | 19.37% |
| **13** | HLA-A*03:01:01:01 | HLA-B*08:01:01 | HLA-C*07:01:01 | HLA-DQA1*01:02:01:01 | HLA-DQB1*03:01:01:03 | HLA-DRB1*15:01:01 | 2.00 | 0.90% | 20.27% |
| **14** | HLA-A*03:01:01:01 | HLA-B*15:01:01:01 | HLA-C*07:04:01 | HLA-DQA1*01:03:01:01 | HLA-DQB1*06:01:01/15 | HLA-DRB1*15:02:01:01 | 2.00 | 0.90% | 21.17% |
| **15** | HLA-A*03:01:01:01 | HLA-B*35:03:01 | HLA-C*04:01:01:01 | HLA-DQA1*03:01:01 | HLA-DQB1*03:02:01:01 | HLA-DRB1*04:03:01:01 | 2.00 | 0.90% | 22.07% |
| **16** | HLA-A*03:01:01:01 | HLA-B*44:27:01 | HLA-C*07:04:01 | HLA-DQA1*01:02:02 | HLA-DQB1*05:02:01:01 | HLA-DRB1*16:01:01 | 2.00 | 0.90% | 22.97% |
| **17** | HLA-A*11:01:01:01 | HLA-B*52:01:01 | HLA-C*12:02:02 | HLA-DQA1*01:04:01:01 | HLA-DQB1*05:03:01 | HLA-DRB1*14:54:01:02 | 2.00 | 0.90% | 23.87% |
| **18** | HLA-A*23:01:01:01 | HLA-B*44:03:01 | HLA-C*04:01:01:01 | HLA-DQA1*02:01:01:01 | HLA-DQB1*02:02:01:01 | HLA-DRB1*07:01:01 | 2.00 | 0.90% | 24.78% |
| **19** | HLA-A*24:02:01:01 | HLA-B*07:02:01 | HLA-C*07:02:01 | HLA-DQA1*01:02:02 | HLA-DQB1*05:02:01:01 | HLA-DRB1*15:01:01 | 2.00 | 0.90% | 25.68% |
| **20** | HLA-A*24:02:01:01 | HLA-B*51:01:01 | HLA-C*15:02:01:01 | HLA-DQA1*01:02:02 | HLA-DQB1*05:02:01:01 | HLA-DRB1*16:01:01 | 2.00 | 0.90% | 26.58% |
| **21** | HLA-A*31:01:02:01 | HLA-B*07:02:01 | HLA-C*07:02:01 | HLA-DQA1*01:02:01:01 | HLA-DQB1*06:02:01:01 | HLA-DRB1*15:01:01 | 2.00 | 0.90% | 27.48% |
| **22** | HLA-A*66:01:01:01 | HLA-B*41:02:01 | HLA-C*17:03:01 | HLA-DQA1*05:05:01:05 | HLA-DQB1*03:01:01:03 | HLA-DRB1*13:03:01 | 2.00 | 0.90% | 28.38% |
| **23** | HLA-A*02:01:01 | HLA-B*35:01:01 | HLA-C*04:01:01:06 | HLA-DQA1*01:03:01:02 | HLA-DQB1*06:03:01:02 | HLA-DRB1*13:01:01:02 | 1.00 | 0.45% | 28.83% |
| **24** | HLA-A*01:01:01:01 | HLA-B*08:01:01 | HLA-C*07:01:01 | HLA-DQA1*01:02:01:04 | HLA-DQB1*06:04:01 | HLA-DRB1*13:02:01:02 | 1.00 | 0.45% | 29.28% |
| **25** | HLA-A*01:01:01:01 | HLA-B*08:01:01 | HLA-C*07:01:01 | HLA-DQA1*05:01:01:02 | HLA-DQB1*02:01:01 | HLA-DRB1*03:01:01:02 | 1.00 | 0.45% | 29.73% |
| **26** | HLA-A*01:01:01:01 | HLA-B*08:01:01 | HLA-C*07:01:01 | HLA-DQA1*05:05:01:01 | HLA-DQB1*03:01:01:03 | HLA-DRB1*11:03:01 | 1.00 | 0.45% | 30.18% |
| **27** | HLA-A*01:01:01:01 | HLA-B*14:02:01:01 | HLA-C*08:02:01:01 | HLA-DQA1*03:01:01 | HLA-DQB1*03:02:01:01 | HLA-DRB1*04:01:01:01 | 1.00 | 0.45% | 30.63% |
| **28** | HLA-A*01:01:01:01 | HLA-B*15:17:01:01 | HLA-C*07:01:02 | HLA-DQA1*01:03:01:02 | HLA-DQB1*06:03:01:01 | HLA-DRB1*13:01:01:01 | 1.00 | 0.45% | 31.08% |
| **29** | HLA-A*01:01:01:01 | HLA-B*18:01:01 | HLA-C*12:03:01 | HLA-DQA1*05:05:01:04 | HLA-DQB1*03:01:01:03 | HLA-DRB1*11:03:01 | 1.00 | 0.45% | 31.53% |
| **30** | HLA-A*01:01:01:01 | HLA-B*27:02:01:01 | HLA-C*02:02:02 | HLA-DQA1*01:02:01:05 | HLA-DQB1*06:02:01:01 | HLA-DRB1*15:01:01 | 1.00 | 0.45% | 31.98% |
| **31** | HLA-A*01:01:01:01 | HLA-B*27:02:01:01 | HLA-C*02:02:02 | HLA-DQA1*03:01:01 | HLA-DQB1*03:02:01:01 | HLA-DRB1*04:01:01:03 | 1.00 | 0.45% | 32.43% |
| **32** | HLA-A*01:01:01:01 | HLA-B*27:05:02 | HLA-C*02:02:02 | HLA-DQA1*05:05:01:09 | HLA-DQB1*06:02:01:01 | HLA-DRB1*11:01:01:01 | 1.00 | 0.45% | 32.88% |
| **33** | HLA-A*01:01:01:01 | HLA-B*35:03:01 | HLA-C*04:01:01:01 | HLA-DQA1*05:05:01:01 | HLA-DQB1*03:01:01:03 | HLA-DRB1*11:01:01:01 | 1.00 | 0.45% | 33.33% |
| **34** | HLA-A*01:01:01:01 | HLA-B*35:08:01:01 | HLA-C*04:01:01:06 | HLA-DQA1*03:01:01 | HLA-DQB1*02:01:01 | HLA-DRB1*03:01:01:03 | 1.00 | 0.45% | 33.78% |
| **35** | HLA-A*01:01:01:01 | HLA-B*50:01:01:01 | HLA-C*07:01:01 | HLA-DQA1*02:01:01:01 | HLA-DQB1*02:02:01:01 | HLA-DRB1*04:02:01 | 1.00 | 0.45% | 34.24% |
| **36** | HLA-A*01:01:01:01 | HLA-B*54:01:01 | HLA-C*06:02:01:01 | HLA-DQA1*01:04:01:02 | HLA-DQB1*05:03:01 | HLA-DRB1*07:01:01 | 1.00 | 0.45% | 34.69% |
| **37** | HLA-A*01:01:01:01 | HLA-B*55:01:01 | HLA-C*01:02:01 | HLA-DQA1*01:04:01:01 | HLA-DQB1*05:03:01 | HLA-DRB1*14:54:01:02 | 1.00 | 0.45% | 35.14% |
| **38** | HLA-A*01:01:01:01 | HLA-B*55:01:01 | HLA-C*03:03:01 | HLA-DQA1*05:05:01:05 | HLA-DQB1*03:02:01:01 | HLA-DRB1*13:03:01 | 1.00 | 0.45% | 35.59% |
| **39** | HLA-A*01:01:01:01 | HLA-B*55:01:01 | HLA-C*03:04:01 | HLA-DQA1*01:02:02 | HLA-DQB1*02:01:01 | HLA-DRB1*16:01:01 | 1.00 | 0.45% | 36.04% |
| **40** | HLA-A*01:01:01:01 | HLA-B*55:01:01 | HLA-C*04:01:01:01 | HLA-DQA1*01:02:01:04 | HLA-DQB1*06:04:01 | HLA-DRB1*13:02:01:02 | 1.00 | 0.45% | 36.49% |
| **41** | HLA-A*01:01:01:01 | HLA-B*55:01:01 | HLA-C*07:01:01 | HLA-DQA1*01:03:01:02 | HLA-DQB1*05:03:01 | HLA-DRB1*14:54:01:02 | 1.00 | 0.45% | 36.94% |
| **42** | HLA-A*02:01:01 | HLA-B*07:02:01 | HLA-C*07:02:01 | HLA-DQA1*01:03:01:02 | HLA-DQB1*05:01:01:05 | HLA-DRB1*10:01:01:01 | 1.00 | 0.45% | 37.39% |
| **43** | HLA-A*02:01:01 | HLA-B*07:02:01 | HLA-C*12:03:01 | HLA-DQA1*01:01:01:03 | HLA-DQB1*05:01:01:03 | HLA-DRB1*01:01:01 | 1.00 | 0.45% | 37.84% |
| **44** | HLA-A*02:01:01 | HLA-B*07:06:01 | HLA-C*15:05:02 | HLA-DQA1*01:02:02 | HLA-DQB1*03:02:01:01 | HLA-DRB1*16:01:01 | 1.00 | 0.45% | 38.29% |
| **45** | HLA-A*02:01:01 | HLA-B*08:01:01 | HLA-C*07:01:01 | HLA-DQA1*05:01:01:02 | HLA-DQB1*02:01:01 | HLA-DRB1*03:01:01:01 | 1.00 | 0.45% | 38.74% |
| **46** | HLA-A*02:01:01 | HLA-B*15:01:01:01 | HLA-C*02:02:02 | HLA-DQA1*01:03:01:02 | HLA-DQB1*06:03:01:01 | HLA-DRB1*11:01:08 | 1.00 | 0.45% | 39.19% |
| **47** | HLA-A*02:01:01 | HLA-B*15:01:06 | HLA-C*03:04:01 | HLA-DQA1*01:03:01:02 | HLA-DQB1*05:01:01:03 | HLA-DRB1*01:01:01 | 1.00 | 0.45% | 39.64% |
| **48** | HLA-A*02:01:01 | HLA-B*18:01:01 | HLA-C*01:02:01 | HLA-DQA1*02:01:01:02 | HLA-DQB1*03:02:01:01 | HLA-DRB1*07:01:01 | 1.00 | 0.45% | 40.09% |
| **49** | HLA-A*02:01:01 | HLA-B*18:01:01 | HLA-C*07:01:09 | HLA-DQA1*01:04:01:02 | HLA-DQB1*05:03:01 | HLA-DRB1*14:01:01 | 1.00 | 0.45% | 40.54% |
| **50** | HLA-A*02:01:01 | HLA-B*18:01:01 | HLA-C*07:02:01 | HLA-DQA1*01:02:02 | HLA-DQB1*05:02:01:01 | HLA-DRB1*16:02:01:02 | 1.00 | 0.45% | 40.99% |
| **51** | HLA-A*02:01:01 | HLA-B*27:02:01:01 | HLA-C*01:02:01 | HLA-DQA1*01:02:02 | HLA-DQB1*05:01:01:03 | HLA-DRB1*16:01:02 | 1.00 | 0.45% | 41.44% |
| **52** | HLA-A*02:01:01 | HLA-B*27:05:02 | HLA-C*02:02:02 | HLA-DQA1*01:01:01:03 | HLA-DQB1*05:02:01:01 | HLA-DRB1*01:01:01 | 1.00 | 0.45% | 41.89% |
| **53** | HLA-A*02:01:01 | HLA-B*27:05:02 | HLA-C*02:02:02 | HLA-DQA1*01:02:01:05 | HLA-DQB1*06:02:01:01 | HLA-DRB1*15:01:01 | 1.00 | 0.45% | 42.34% |
| **54** | HLA-A*02:01:01 | HLA-B*27:05:02 | HLA-C*02:02:02 | HLA-DQA1*01:03:01:02 | HLA-DQB1*06:03:01:01 | HLA-DRB1*13:01:01:01 | 1.00 | 0.45% | 42.79% |
| **55** | HLA-A*02:01:01 | HLA-B*27:05:02 | HLA-C*07:02:01 | HLA-DQA1*01:01:01:01 | HLA-DQB1*05:01:01:03 | HLA-DRB1*01:01:01 | 1.00 | 0.45% | 43.25% |
| **56** | HLA-A*02:01:01 | HLA-B*27:05:02 | HLA-C*07:02:01 | HLA-DQA1*01:01:01:03 | HLA-DQB1*03:01:01:03 | HLA-DRB1*01:01:01 | 1.00 | 0.45% | 43.70% |
| **57** | HLA-A*02:01:01 | HLA-B*27:05:02 | HLA-C*12:03:01 | HLA-DQA1*01:02:01:01 | HLA-DQB1*06:02:01:01 | HLA-DRB1*15:01:01 | 1.00 | 0.45% | 44.15% |
| **58** | HLA-A*02:01:01 | HLA-B*35:01:01 | HLA-C*02:02:02 | HLA-DQA1*01:01:01:01 | HLA-DQB1*03:01:01:03 | HLA-DRB1*01:01:01 | 1.00 | 0.45% | 44.60% |
| **59** | HLA-A*02:01:01 | HLA-B*35:01:01 | HLA-C*07:02:01 | HLA-DQA1*01:04:01:04 | HLA-DQB1*03:01:01:03 | HLA-DRB1*11:01:01:03 | 1.00 | 0.45% | 45.05% |
| **60** | HLA-A*02:01:01 | HLA-B*35:02:01 | HLA-C*01:02:01 | HLA-DQA1*01:01:01:03 | HLA-DQB1*05:01:01:03 | HLA-DRB1*01:01:01 | 1.00 | 0.45% | 45.50% |
| **61** | HLA-A*02:01:01 | HLA-B*35:03:01 | HLA-C*04:01:01:01 | HLA-DQA1*01:01:02 | HLA-DQB1*05:01:01:01 | HLA-DRB1*01:02:01 | 1.00 | 0.45% | 45.95% |
| **62** | HLA-A*02:01:01 | HLA-B*35:03:01 | HLA-C*05:01:01 | HLA-DQA1*01:04:01:01 | HLA-DQB1*05:03:01 | HLA-DRB1*14:54:01:02 | 1.00 | 0.45% | 46.40% |
| **63** | HLA-A*02:01:01 | HLA-B*35:03:01 | HLA-C*07:04:01 | HLA-DQA1*01:04:01:02 | HLA-DQB1*03:02:01:01 | HLA-DRB1*14:54:01:02 | 1.00 | 0.45% | 46.85% |
| **64** | HLA-A*02:01:01 | HLA-B*35:03:01 | HLA-C*14:02:01 | HLA-DQA1*01:02:01:01 | HLA-DQB1*06:02:01:01 | HLA-DRB1*15:01:01 | 1.00 | 0.45% | 47.30% |
| **65** | HLA-A*02:01:01 | HLA-B*37:01:01:01 | HLA-C*06:02:01:01 | HLA-DQA1*01:05:01 | HLA-DQB1*05:01:01:05 | HLA-DRB1*10:01:01:03 | 1.00 | 0.45% | 47.75% |
| **66** | HLA-A*02:01:01 | HLA-B*38:01:01 | HLA-C*12:03:01 | HLA-DQA1*01:01:01:05 | HLA-DQB1*05:01:01:03 | HLA-DRB1*01:01:01 | 1.00 | 0.45% | 48.20% |
| **67** | HLA-A*02:01:01 | HLA-B*38:01:01 | HLA-C*14:02:01 | HLA-DQA1*04:01:01:03 | HLA-DQB1*04:02:01:01 | HLA-DRB1*13:01:01:02 | 1.00 | 0.45% | 48.65% |
| **68** | HLA-A*02:01:01 | HLA-B*39:01:01 | HLA-C*05:01:01 | HLA-DQA1*05:05:01:04 | HLA-DQB1*03:01:01:03 | HLA-DRB1*11:01:01:01 | 1.00 | 0.45% | 49.10% |
| **69** | HLA-A*02:01:01 | HLA-B*39:01:01 | HLA-C*07:02:01 | HLA-DQA1*05:05:01:03 | HLA-DQB1*03:01:01:05 | HLA-DRB1*12:01:01/12:10 | 1.00 | 0.45% | 49.55% |
| **70** | HLA-A*02:01:01 | HLA-B*40:01:02 | HLA-C*01:02:01 | HLA-DQA1*05:01:01:03 | HLA-DQB1*05:02:01:01 | HLA-DRB1*03:01:01:03 | 1.00 | 0.45% | 50.00% |
| **71** | HLA-A*02:01:01 | HLA-B*44:02:01:01 | HLA-C*05:01:01 | HLA-DQA1*01:05:01 | HLA-DQB1*06:03:01:01 | HLA-DRB1*13:01:01:02 | 1.00 | 0.45% | 50.45% |
| **72** | HLA-A*02:01:01 | HLA-B*44:02:01:01 | HLA-C*05:01:01 | HLA-DQA1*03:03:01:01 | HLA-DQB1*05:02:01:01 | HLA-DRB1*04:05:01:03 | 1.00 | 0.45% | 50.90% |
| **73** | HLA-A*02:01:01 | HLA-B*44:02:01:01 | HLA-C*05:01:01 | HLA-DQA1*05:05:01:01 | HLA-DQB1*03:02:01:01 | HLA-DRB1*04:02:01 | 1.00 | 0.45% | 51.35% |
| **74** | HLA-A*02:01:01 | HLA-B*44:05:01 | HLA-C*02:02:02 | HLA-DQA1*01:01:01:05 | HLA-DQB1*05:01:01:03 | HLA-DRB1*01:01:01 | 1.00 | 0.45% | 51.80% |
| **75** | HLA-A*02:01:01 | HLA-B*44:27:01 | HLA-C*15:02:01:01 | HLA-DQA1*05:05:01:09 | HLA-DQB1*03:03:02:02 | HLA-DRB1*11:01:01:01 | 1.00 | 0.45% | 52.26% |
| **76** | HLA-A*02:01:01 | HLA-B*51:01:01 | HLA-C*02:02:02 | HLA-DQA1*03:01:01 | HLA-DQB1*03:02:01:01 | HLA-DRB1*04:15 | 1.00 | 0.45% | 52.71% |
| **77** | HLA-A*02:01:01 | HLA-B*51:01:01 | HLA-C*02:02:02 | HLA-DQA1*05:05:01:04 | HLA-DQB1*05:01:01:03 | HLA-DRB1*11:01:01:03 | 1.00 | 0.45% | 53.16% |
| **78** | HLA-A*02:01:01 | HLA-B*51:01:01 | HLA-C*03:03:01 | HLA-DQA1*05:05:01:04 | HLA-DQB1*03:01:01:19 | HLA-DRB1*13:01:01:01 | 1.00 | 0.45% | 53.61% |
| **79** | HLA-A*02:01:01 | HLA-B*51:01:01 | HLA-C*07:02:01 | HLA-DQA1*01:02:01:01 | HLA-DQB1*06:02:01:01 | HLA-DRB1*11:01:01:01 | 1.00 | 0.45% | 54.06% |
| **80** | HLA-A*02:01:01 | HLA-B*51:01:01 | HLA-C*12:03:01 | HLA-DQA1*01:03:01:02 | HLA-DQB1*06:03:01:01 | HLA-DRB1*08:01:01 | 1.00 | 0.45% | 54.51% |
| **81** | HLA-A*02:01:01 | HLA-B*51:01:01 | HLA-C*12:03:01 | HLA-DQA1*03:01:01 | HLA-DQB1*03:03:02:01 | HLA-DRB1*04:01:01:01 | 1.00 | 0.45% | 54.96% |
| **82** | HLA-A*02:01:01 | HLA-B*51:01:01 | HLA-C*12:03:01 | HLA-DQA1*05:05:01:09 | HLA-DQB1*03:01:01:03 | HLA-DRB1*11:01:01:03 | 1.00 | 0.45% | 55.41% |
| **83** | HLA-A*02:01:01 | HLA-B*51:01:01 | HLA-C*16:02:01 | HLA-DQA1*05:05:01:05 | HLA-DQB1*03:01:01:05 | HLA-DRB1*12:01:01/12:10 | 1.00 | 0.45% | 55.86% |
| **84** | HLA-A*02:01:01 | HLA-B*55:01:01 | HLA-C*03:03:01 | HLA-DQA1*01:02:02 | HLA-DQB1*05:02:01:01 | HLA-DRB1*16:01:01 | 1.00 | 0.45% | 56.31% |
| **85** | HLA-A*02:01:01 | HLA-B*55:01:01 | HLA-C*03:03:01 | HLA-DQA1*01:04:01:02 | HLA-DQB1*05:03:01 | HLA-DRB1*14:54:01:01 | 1.00 | 0.45% | 56.76% |
| **86** | HLA-A*02:01:01 | HLA-B*57:01:01:01 | HLA-C*01:02:01 | HLA-DQA1*04:01:01:03 | HLA-DQB1*03:01:01:03 | HLA-DRB1*08:01:01 | 1.00 | 0.45% | 57.21% |
| **87** | HLA-A*02:01:01 | HLA-B*57:01:01:01 | HLA-C*06:02:01:01 | HLA-DQA1*01:02:01:05 | HLA-DQB1*06:02:01:01 | HLA-DRB1*15:01:01 | 1.00 | 0.45% | 57.66% |
| **88** | HLA-A*02:01:01 | HLA-B*57:01:01:01 | HLA-C*06:02:01:01 | HLA-DQA1*03:01:01 | HLA-DQB1*03:02:01:01 | HLA-DRB1*04:01:01:02 | 1.00 | 0.45% | 58.11% |
| **89** | HLA-A*02:01:01 | HLA-B*57:01:01:01 | HLA-C*06:02:01:01 | HLA-DQA1*05:05:01:04 | HLA-DQB1*03:01:01:03 | HLA-DRB1*11:01:01:01 | 1.00 | 0.45% | 58.56% |
| **90** | HLA-A*03:01:01:01 | HLA-B*07:02:01 | HLA-C*02:02:02 | HLA-DQA1*02:01:01:02 | HLA-DQB1*03:03:02:01 | HLA-DRB1*07:01:01 | 1.00 | 0.45% | 59.01% |
| **91** | HLA-A*03:01:01:01 | HLA-B*07:02:01 | HLA-C*04:01:01:06 | HLA-DQA1*05:05:01:01 | HLA-DQB1*05:03:01 | HLA-DRB1*14:01:01 | 1.00 | 0.45% | 59.46% |
| **92** | HLA-A*03:01:01:01 | HLA-B*14:02:01:01 | HLA-C*08:02:01:01 | HLA-DQA1*05:05:01:06 | HLA-DQB1*03:01:01:03 | HLA-DRB1*04:05:01:03 | 1.00 | 0.45% | 59.91% |
| **93** | HLA-A*03:01:01:01 | HLA-B*15:01:01:01 | HLA-C*03:03:01 | HLA-DQA1*01:02:02 | HLA-DQB1*05:02:01:01 | HLA-DRB1*16:01:01 | 1.00 | 0.45% | 60.36% |
| **94** | HLA-A*03:01:01:01 | HLA-B*15:01:01:01 | HLA-C*07:04:01 | HLA-DQA1*05:01:01:02 | HLA-DQB1*02:01:01 | HLA-DRB1*14:54:01:02 | 1.00 | 0.45% | 60.81% |
| **95** | HLA-A*03:01:01:01 | HLA-B*18:01:01 | HLA-C*12:03:01 | HLA-DQA1*01:05:01 | HLA-DQB1*05:01:01:05 | HLA-DRB1*10:01:01:03 | 1.00 | 0.45% | 61.27% |
| **96** | HLA-A*03:01:01:01 | HLA-B*35:01:01 | HLA-C*04:01:01:01 | HLA-DQA1*01:03:01:02 | HLA-DQB1*06:03:01:01 | HLA-DRB1*13:01:01:02 | 1.00 | 0.45% | 61.72% |
| **97** | HLA-A*03:01:01:01 | HLA-B*35:03:01 | HLA-C*04:01:01:01 | HLA-DQA1*03:01:01 | HLA-DQB1*03:02:01:01 | HLA-DRB1*04:01:01:03 | 1.00 | 0.45% | 62.17% |
| **98** | HLA-A*03:01:01:01 | HLA-B*38:01:01 | HLA-C*05:01:01 | HLA-DQA1*01:02:01:01 | HLA-DQB1*03:01:01:03 | HLA-DRB1*11:01:01:03 | 1.00 | 0.45% | 62.62% |
| **99** | HLA-A*03:01:01:01 | HLA-B*40:02:01 | HLA-C*02:02:02 | HLA-DQA1*02:01:01:02 | HLA-DQB1*03:03:02:01 | HLA-DRB1*07:01:01 | 1.00 | 0.45% | 63.07% |
| **100** | HLA-A*03:01:01:01 | HLA-B*44:02:01:01 | HLA-C*05:01:01 | HLA-DQA1*03:03:01:01 | HLA-DQB1*03:01:01:10 | HLA-DRB1*04:08:01 | 1.00 | 0.45% | 63.52% |
| **101** | HLA-A*03:01:01:01 | HLA-B*44:03:01 | HLA-C*16:01:01 | HLA-DQA1*01:01:01:01 | HLA-DQB1*05:01:01:03 | HLA-DRB1*01:01:01 | 1.00 | 0.45% | 63.97% |
| **102** | HLA-A*03:01:01:01 | HLA-B*49:01:01 | HLA-C*07:01:01 | HLA-DQA1*05:05:01:09 | HLA-DQB1*03:01:01:03 | HLA-DRB1*11:01:01:03 | 1.00 | 0.45% | 64.42% |
| **103** | HLA-A*03:01:01:01 | HLA-B*51:01:01 | HLA-C*15:02:01:01 | HLA-DQA1*03:02:01:01 | HLA-DQB1*03:03:02:02 | HLA-DRB1*09:01:02 | 1.00 | 0.45% | 64.87% |
| **104** | HLA-A*03:01:01:03 | HLA-B*51:01:01 | HLA-C*01:02:01 | HLA-DQA1*05:05:01:02 | HLA-DQB1*03:01:01:03 | HLA-DRB1*11:01:01:01 | 1.00 | 0.45% | 65.32% |
| **105** | HLA-A*03:01:01:05 | HLA-B*55:01:01 | HLA-C*03:03:01 | HLA-DQA1*01:04:01:02 | HLA-DQB1*02:01:01 | HLA-DRB1*03:01:01:03 | 1.00 | 0.45% | 65.77% |
| **106** | HLA-A*11:01:01:01 | HLA-B*07:02:01 | HLA-C*02:02:02 | HLA-DQA1*01:01:01:01 | HLA-DQB1*05:01:01:03 | HLA-DRB1*16:02:01:02 | 1.00 | 0.45% | 66.22% |
| **107** | HLA-A*11:01:01:01 | HLA-B*07:05:01 | HLA-C*15:05:02 | HLA-DQA1*01:05:01 | HLA-DQB1*05:01:01:02 | HLA-DRB1*10:01:01:03 | 1.00 | 0.45% | 66.67% |
| **108** | HLA-A*11:01:01:01 | HLA-B*15:01:01:01 | HLA-C*03:03:01 | HLA-DQA1*01:01:01:02 | HLA-DQB1*05:01:01:03 | HLA-DRB1*01:01:01 | 1.00 | 0.45% | 67.12% |
| **109** | HLA-A*11:01:01:01 | HLA-B*18:01:01 | HLA-C*05:01:01 | HLA-DQA1*01:02:01:01 | HLA-DQB1*02:01:01 | HLA-DRB1*15:01:01 | 1.00 | 0.45% | 67.57% |
| **110** | HLA-A*11:01:01:01 | HLA-B*18:03 | HLA-C*07:01:01 | HLA-DQA1*05:05:01:04 | HLA-DQB1*06:03:01:01 | HLA-DRB1*11:04:01 | 1.00 | 0.45% | 68.02% |
| **111** | HLA-A*11:01:01:01 | HLA-B*35:01:01 | HLA-C*05:01:01 | HLA-DQA1*05:05:01:06 | HLA-DQB1*05:03:01 | HLA-DRB1*12:01:01/12:10 | 1.00 | 0.45% | 68.47% |
| **112** | HLA-A*11:01:01:01 | HLA-B*35:03:01 | HLA-C*03:03:01 | HLA-DQA1*02:01:01:02 | HLA-DQB1*02:02:01:01 | HLA-DRB1*07:01:01 | 1.00 | 0.45% | 68.92% |
| **113** | HLA-A*11:01:01:01 | HLA-B*35:03:01 | HLA-C*06:02:01:01 | HLA-DQA1*03:01:01 | HLA-DQB1*05:02:01:01 | HLA-DRB1*08:01:01 | 1.00 | 0.45% | 69.37% |
| **114** | HLA-A*11:01:01:01 | HLA-B*51:01:01 | HLA-C*04:01:01:01 | HLA-DQA1*01:01:01:02 | HLA-DQB1*05:01:01:03 | HLA-DRB1*01:01:01 | 1.00 | 0.45% | 69.82% |
| **115** | HLA-A*11:01:01:01 | HLA-B*51:01:01 | HLA-C*15:02:01:01 | HLA-DQA1*05:05:01:08 | HLA-DQB1*06:02:01:01 | HLA-DRB1*11:01:01:03 | 1.00 | 0.45% | 70.28% |
| **116** | HLA-A*11:01:01:01 | HLA-B*52:01:01 | HLA-C*12:02:02 | HLA-DQA1*01:02:01:03 | HLA-DQB1*05:04 | HLA-DRB1*01:01:01 | 1.00 | 0.45% | 70.73% |
| **117** | HLA-A*11:01:01:01 | HLA-B*52:01:01 | HLA-C*12:02:02 | HLA-DQA1*01:03:01:01 | HLA-DQB1*06:01:01/15 | HLA-DRB1*15:02:01:01 | 1.00 | 0.45% | 71.18% |
| **118** | HLA-A*11:01:01:01 | HLA-B*57:01:01:01 | HLA-C*04:01:01:01 | HLA-DQA1*01:02:02 | HLA-DQB1*03:02:01:01 | HLA-DRB1*16:01:01 | 1.00 | 0.45% | 71.63% |
| **119** | HLA-A*23:01:01:01 | HLA-B*18:03 | HLA-C*04:01:01:01 | HLA-DQA1*05:05:01:04 | HLA-DQB1*03:01:01:03 | HLA-DRB1*11:04:01 | 1.00 | 0.45% | 72.08% |
| **120** | HLA-A*23:01:01:01 | HLA-B*44:02:01:01 | HLA-C*05:01:01 | HLA-DQA1*01:03:01:02 | HLA-DQB1*06:03:01:01 | HLA-DRB1*13:01:01:01 | 1.00 | 0.45% | 72.53% |
| **121** | HLA-A*23:01:01:01 | HLA-B*44:03:01 | HLA-C*04:01:01:01 | HLA-DQA1*02:01:01:02 | HLA-DQB1*02:02:01:01 | HLA-DRB1*07:01:01 | 1.00 | 0.45% | 72.98% |
| **122** | HLA-A*24:02:01:01 | HLA-B*07:02:01 | HLA-C*01:02:01 | HLA-DQA1*05:05:01:02 | HLA-DQB1*03:01:01:03 | HLA-DRB1*15:01:01 | 1.00 | 0.45% | 73.43% |
| **123** | HLA-A*24:02:01:01 | HLA-B*08:01:01 | HLA-C*07:02:01 | HLA-DQA1*05:01:01:03 | HLA-DQB1*02:01:01 | HLA-DRB1*03:01:01:01 | 1.00 | 0.45% | 73.88% |
| **124** | HLA-A*24:02:01:01 | HLA-B*15:10:01 | HLA-C*03:03:01 | HLA-DQA1*01:02:01:04 | HLA-DQB1*06:03:01:02 | HLA-DRB1*13:02:01:02 | 1.00 | 0.45% | 74.33% |
| **125** | HLA-A*24:02:01:01 | HLA-B*15:17:01:01 | HLA-C*07:01:02 | HLA-DQA1*01:01:01:03 | HLA-DQB1*05:01:01:03 | HLA-DRB1*01:01:01 | 1.00 | 0.45% | 74.78% |
| **126** | HLA-A*24:02:01:01 | HLA-B*18:01:01 | HLA-C*03:04:01 | HLA-DQA1*05:05:01:01 | HLA-DQB1*06:04:01 | HLA-DRB1*13:02:01:02 | 1.00 | 0.45% | 75.23% |
| **127** | HLA-A*24:02:01:01 | HLA-B*27:05:02 | HLA-C*04:01:01:06 | HLA-DQA1*05:05:01:09 | HLA-DQB1*03:01:01:03 | HLA-DRB1*11:04:01 | 1.00 | 0.45% | 75.68% |
| **128** | HLA-A*24:02:01:01 | HLA-B*35:01:01 | HLA-C*04:01:01:01 | HLA-DQA1*03:01:01 | HLA-DQB1*03:01:01:03 | HLA-DRB1*11:04:01 | 1.00 | 0.45% | 76.13% |
| **129** | HLA-A*24:02:01:01 | HLA-B*35:02:01 | HLA-C*04:01:01:06 | HLA-DQA1*05:05:01:01 | HLA-DQB1*03:01:01:02 | HLA-DRB1*11:04:01 | 1.00 | 0.45% | 76.58% |
| **130** | HLA-A*24:02:01:01 | HLA-B*35:02:01 | HLA-C*04:01:01:06 | HLA-DQA1*05:05:01:02 | HLA-DQB1*05:01:01:03 | HLA-DRB1*13:05:01 | 1.00 | 0.45% | 77.03% |
| **131** | HLA-A*24:02:01:01 | HLA-B*35:02:01 | HLA-C*04:01:01:06 | HLA-DQA1*05:05:01:04 | HLA-DQB1*03:01:01:03 | HLA-DRB1*11:01:01:01 | 1.00 | 0.45% | 77.48% |
| **132** | HLA-A*24:02:01:01 | HLA-B*35:03:01 | HLA-C*04:01:01:01 | HLA-DQA1*02:01:01:01 | HLA-DQB1*02:02:01:01 | HLA-DRB1*07:01:01 | 1.00 | 0.45% | 77.93% |
| **133** | HLA-A*24:02:01:01 | HLA-B*39:01:01 | HLA-C*07:02:01 | HLA-DQA1*04:01:01:03 | HLA-DQB1*04:02:01:01 | HLA-DRB1*08:01:01 | 1.00 | 0.45% | 78.38% |
| **134** | HLA-A*24:02:01:01 | HLA-B*40:01:02 | HLA-C*03:03:01 | HLA-DQA1*01:01:01:03 | HLA-DQB1*06:03:01:01 | HLA-DRB1*13:01:01:01 | 1.00 | 0.45% | 78.83% |
| **135** | HLA-A*24:02:01:01 | HLA-B*40:01:02 | HLA-C*07:01:01 | HLA-DQA1*01:02:01:04 | HLA-DQB1*03:01:01:03 | HLA-DRB1*11:04:01 | 1.00 | 0.45% | 79.29% |
| **136** | HLA-A*24:02:01:01 | HLA-B*41:02:01 | HLA-C*12:03:01 | HLA-DQA1*03:01:01 | HLA-DQB1*03:01:01:03 | HLA-DRB1*04:01:01:03 | 1.00 | 0.45% | 79.74% |
| **137** | HLA-A*24:02:01:01 | HLA-B*49:01:01 | HLA-C*07:01:01 | HLA-DQA1*04:01:01:03 | HLA-DQB1*04:02:01:01 | HLA-DRB1*08:01:01 | 1.00 | 0.45% | 80.19% |
| **138** | HLA-A*24:02:01:01 | HLA-B*51:01:01 | HLA-C*01:02:01 | HLA-DQA1*01:01:01:01 | HLA-DQB1*05:01:01:03 | HLA-DRB1*01:01:01 | 1.00 | 0.45% | 80.64% |
| **139** | HLA-A*24:02:01:01 | HLA-B*51:01:01 | HLA-C*07:04:01 | HLA-DQA1*03:02:01:01 | HLA-DQB1*03:01:01:03 | HLA-DRB1*09:01:02 | 1.00 | 0.45% | 81.09% |
| **140** | HLA-A*24:02:01:01 | HLA-B*52:01:01 | HLA-C*12:02:02 | HLA-DQA1*01:03:01:01 | HLA-DQB1*06:03:01:01 | HLA-DRB1*13:01:01:02 | 1.00 | 0.45% | 81.54% |
| **141** | HLA-A*24:02:01:05 | HLA-B*15:01:01:01 | HLA-C*03:03:01 | HLA-DQA1*02:01:01:02 | HLA-DQB1*03:03:02:01 | HLA-DRB1*07:01:01 | 1.00 | 0.45% | 81.99% |
| **142** | HLA-A*24:02:01:05 | HLA-B*27:05:02 | HLA-C*02:02:02 | HLA-DQA1*01:03:01:02 | HLA-DQB1*03:01:01:03 | HLA-DRB1*13:01:01:01 | 1.00 | 0.45% | 82.44% |
| **143** | HLA-A*25:01:01:01 | HLA-B*08:01:01 | HLA-C*03:04:01 | HLA-DQA1*01:04:01:01 | HLA-DQB1*06:03:01:01 | HLA-DRB1*13:01:01:01 | 1.00 | 0.45% | 82.89% |
| **144** | HLA-A*25:01:01:01 | HLA-B*15:01:01:01 | HLA-C*03:03:01 | HLA-DQA1*03:03:01:01 | HLA-DQB1*03:01:01:01 | HLA-DRB1*04:08:01 | 1.00 | 0.45% | 83.34% |
| **145** | HLA-A*25:01:01:01 | HLA-B*35:03:01 | HLA-C*04:01:01:01 | HLA-DQA1*01:01:01:02 | HLA-DQB1*05:01:01:03 | HLA-DRB1*01:01:01 | 1.00 | 0.45% | 83.79% |
| **146** | HLA-A*25:01:01:01 | HLA-B*35:08:01:01 | HLA-C*12:03:01 | HLA-DQA1*05:01:01:02 | HLA-DQB1*06:02:01:01 | HLA-DRB1*03:01:01:01 | 1.00 | 0.45% | 84.24% |
| **147** | HLA-A*26:01:01:01 | HLA-B*07:02:01 | HLA-C*12:03:01 | HLA-DQA1*01:03:01:06 | HLA-DQB1*05:02:01:01 | HLA-DRB1*13:01:01:02 | 1.00 | 0.45% | 84.69% |
| **148** | HLA-A*26:01:01:01 | HLA-B*27:02:01:04 | HLA-C*12:03:01 | HLA-DQA1*05:05:01:09 | HLA-DQB1*06:02:01:01 | HLA-DRB1*15:01:01 | 1.00 | 0.45% | 85.14% |
| **149** | HLA-A*26:01:01:01 | HLA-B*51:01:01 | HLA-C*15:02:01:01 | HLA-DQA1*05:01:01:03 | HLA-DQB1*05:03:01 | HLA-DRB1*14:54:01:02 | 1.00 | 0.45% | 85.59% |
| **150** | HLA-A*26:01:01:01 | HLA-B*56:01:01 | HLA-C*06:02:01:01 | HLA-DQA1*05:05:01:08 | HLA-DQB1*04:02:01:01 | HLA-DRB1*11:01:01:01 | 1.00 | 0.45% | 86.04% |
| **151** | HLA-A*26:01:01:06 | HLA-B*51:01:01 | HLA-C*14:02:01 | HLA-DQA1*04:02 | HLA-DQB1*04:02:01:01 | HLA-DRB1*08:01:01 | 1.00 | 0.45% | 86.49% |
| **152** | HLA-A*26:08 | HLA-B*35:01:01 | HLA-C*04:01:01:06 | HLA-DQA1*03:01:01 | HLA-DQB1*03:02:01:01 | HLA-DRB1*04:02:01 | 1.00 | 0.45% | 86.94% |
| **153** | HLA-A*29:01:01:01 | HLA-B*08:01:01 | HLA-C*06:02:01:02 | HLA-DQA1*03:01:01 | HLA-DQB1*03:02:01:01 | HLA-DRB1*07:01:01 | 1.00 | 0.45% | 87.39% |
| **154** | HLA-A*29:02:01:01 | HLA-B*56:01:01 | HLA-C*01:02:01 | HLA-DQA1*02:01:01:02 | HLA-DQB1*02:02:01:01 | HLA-DRB1*07:01:01 | 1.00 | 0.45% | 87.84% |
| **155** | HLA-A*29:02:01:01 | HLA-B*58:01:01 | HLA-C*07:18 | HLA-DQA1*04:01:02:01 | HLA-DQB1*04:02:01:01 | HLA-DRB1*08:04:01 | 1.00 | 0.45% | 88.30% |
| **156** | HLA-A*30:01:01 | HLA-B*15:01:01:01 | HLA-C*03:04:02 | HLA-DQA1*05:05:01:04 | HLA-DQB1*03:01:01:03 | HLA-DRB1*11:03:01 | 1.00 | 0.45% | 88.75% |
| **157** | HLA-A*30:01:01 | HLA-B*35:01:01 | HLA-C*04:01:01:01 | HLA-DQA1*01:01:01:02 | HLA-DQB1*05:01:01:03 | HLA-DRB1*01:01:01 | 1.00 | 0.45% | 89.20% |
| **158** | HLA-A*30:01:01 | HLA-B*35:03:01 | HLA-C*12:03:01 | HLA-DQA1*01:04:01:03 | HLA-DQB1*05:03:01 | HLA-DRB1*03:01:01:01 | 1.00 | 0.45% | 89.65% |
| **159** | HLA-A*31:01:02:01 | HLA-B*15:17:01:01 | HLA-C*07:01:02 | HLA-DQA1*01:01:01:05 | HLA-DQB1*05:01:01:03 | HLA-DRB1*01:01:01 | 1.00 | 0.45% | 90.10% |
| **160** | HLA-A*31:01:02:01 | HLA-B*37:01:01:01 | HLA-C*01:02:01 | HLA-DQA1*02:01:01:01 | HLA-DQB1*02:02:01:01 | HLA-DRB1*14:05:01:02 | 1.00 | 0.45% | 90.55% |
| **161** | HLA-A*31:01:02:01 | HLA-B*58:01:01 | HLA-C*07:18 | HLA-DQA1*05:01:01:03 | HLA-DQB1*03:02:01:02 | HLA-DRB1*04:04:01 | 1.00 | 0.45% | 91.00% |
| **162** | HLA-A*31:01:02:04 | HLA-B*27:02:01:01 | HLA-C*02:02:02 | HLA-DQA1*01:02:02 | HLA-DQB1*06:03:01:01 | HLA-DRB1*16:01:01 | 1.00 | 0.45% | 91.45% |
| **163** | HLA-A*32:01:01:01 | HLA-B*18:01:01 | HLA-C*03:04:01 | HLA-DQA1*01:01:01:03 | HLA-DQB1*05:01:01:03 | HLA-DRB1*01:01:01 | 1.00 | 0.45% | 91.90% |
| **164** | HLA-A*32:01:01:01 | HLA-B*35:01:01 | HLA-C*04:01:01:01 | HLA-DQA1*01:03:01:02 | HLA-DQB1*06:03:01:01 | HLA-DRB1*13:01:01:02 | 1.00 | 0.45% | 92.35% |
| **165** | HLA-A*32:01:01:01 | HLA-B*38:01:01 | HLA-C*12:03:01 | HLA-DQA1*01:10 | HLA-DQB1*06:01:01/15 | HLA-DRB1*15:02:01:01 | 1.00 | 0.45% | 92.80% |
| **166** | HLA-A*32:01:01:01 | HLA-B*40:02:01 | HLA-C*02:02:02 | HLA-DQA1*02:01:01:01 | HLA-DQB1*02:02:01:01 | HLA-DRB1*07:01:01 | 1.00 | 0.45% | 93.25% |
| **167** | HLA-A*32:01:01:01 | HLA-B*40:02:01 | HLA-C*07:02:01 | HLA-DQA1*01:02:02 | HLA-DQB1*05:02:01:01 | HLA-DRB1*01:01:01 | 1.00 | 0.45% | 93.70% |
| **168** | HLA-A*32:01:01:01 | HLA-B*44:02:01:01 | HLA-C*04:01:01:01 | HLA-DQA1*01:04:01:01 | HLA-DQB1*03:01:01:05 | HLA-DRB1*14:54:01:02 | 1.00 | 0.45% | 94.15% |
| **169** | HLA-A*32:01:01:01 | HLA-B*44:02:01:01 | HLA-C*04:01:01:01 | HLA-DQA1*02:01:01:01 | HLA-DQB1*03:03:02:01 | HLA-DRB1*07:01:01 | 1.00 | 0.45% | 94.60% |
| **170** | HLA-A*32:01:01:01 | HLA-B*44:02:01:01 | HLA-C*12:03:01 | HLA-DQA1*01:02:02 | HLA-DQB1*05:02:01:01 | HLA-DRB1*16:01:01 | 1.00 | 0.45% | 95.05% |
| **171** | HLA-A*33:01:01:01 | HLA-B*14:02:01:01 | HLA-C*08:02:01:01 | HLA-DQA1*05:05:01:02 | HLA-DQB1*03:01:01:03 | HLA-DRB1*11:01:01:01 | 1.00 | 0.45% | 95.50% |
| **172** | HLA-A*33:03:01:02 | HLA-B*58:01:01 | HLA-C*03:02:02:01 | HLA-DQA1*05:01:01:03 | HLA-DQB1*02:01:01 | HLA-DRB1*03:01:01:03 | 1.00 | 0.45% | 95.95% |
| **173** | HLA-A*68:01:01:02 | HLA-B*44:02:01:01 | HLA-C*05:01:01 | HLA-DQA1*03:01:01 | HLA-DQB1*03:02:01:01 | HLA-DRB1*08:01:01 | 1.00 | 0.45% | 96.40% |
| **174** | HLA-A*68:01:02:02 | HLA-B*35:01:01 | HLA-C*04:01:01:01 | HLA-DQA1*03:01:01 | HLA-DQB1*05:03:01 | HLA-DRB1*04:03:01:01 | 1.00 | 0.45% | 96.85% |
| **175** | HLA-A*68:01:02:02 | HLA-B*44:03:01 | HLA-C*04:01:01:01 | HLA-DQA1*01:02:02 | HLA-DQB1*05:02:01:01 | HLA-DRB1*16:01:01 | 1.00 | 0.45% | 97.31% |
| **176** | HLA-A*68:01:02:02 | HLA-B*51:01:01 | HLA-C*15:02:01:01 | HLA-DQA1*04:02 | HLA-DQB1*04:02:01:01 | HLA-DRB1*08:01:01 | 1.00 | 0.45% | 97.76% |
| **177** | HLA-A*68:02:01:01 | HLA-B*39:01:01 | HLA-C*12:03:01 | HLA-DQA1*01:02:02 | HLA-DQB1*05:02:01:01 | HLA-DRB1*16:01:01 | 1.00 | 0.45% | 98.21% |
| **178** | HLA-A*01:01:01:01 | HLA-B*08:01:01 | HLA-C*07:01:02 | HLA-DQA1*01:02:01:04 | HLA-DQB1*06:02:01:01 | HLA-DRB1*13:02:01:02 | 1.00 | 0.45% | 98.66% |
| **179** | HLA-A*02:01:01 | HLA-B*15:17:01:01 | HLA-C*07:01:01 | HLA-DQA1*01:02:01:01 | HLA-DQB1*06:04:01 | HLA-DRB1*15:01:01 | 1.00 | 0.45% | 99.11% |
| **180** | HLA-A*02:01:01 | HLA-B*44:02:01:03 | HLA-C*04:01:01:06 | HLA-DQA1*01:01:01:01 | HLA-DQB1*06:03:01:02 | HLA-DRB1*01:01:01 | 1.00 | 0.45% | 99.56% |
| **181** | HLA-A*25:01:01:01 | HLA-B*35:01:01 | HLA-C*07:04:01 | HLA-DQA1*01:03:01:02 | HLA-DQB1*05:01:01:03 | HLA-DRB1*13:01:01:02 | 1.00 | 0.45% | 100.0% |
